# Alkali-Metal Interlocking of 2D V_4_O_10_ Sheets Defines Discretized Interlayer Shear Relationships

**DOI:** 10.1021/jacs.5c16903

**Published:** 2026-02-19

**Authors:** John Ponis, Kenna Ashen, Sarbajeet Chakraborty, George Agbeworvi, Michelle A. Smeaton, Chengdong Wang, Amanda Jessel, Douglas H. Fabini, Fanni Juranyi, Diana Quintero-Castro, Nick A. Shepelin, Dariusz Jakub Gawryluk, Katherine L. Jungjohann, Shruti Hariyani, Xiaofeng Qian, Sarbajit Banerjee

**Affiliations:** † Department of Chemistry, 14736 Texas A&M University, College Station, Texas 77843, United States; ‡ Department of Materials Science and Engineering, Texas A&M University, College Station, Texas 77843, United States; § Laboratory for Battery Science, PSI Center for Energy and Environmental Sciences, 28498 Paul Scherrer Institute, Forschungsstrasse 111, CH-5232 Villigen PSI, Switzerland; ∥ Laboratory for Inorganic Chemistry, Department of Chemistry and Applied Biosciences, 27219 ETH Zurich, Vladimir-Prelog-Weg 2, CH-8093 Zürich, Switzerland; ⊥ National Laboratory of the Rockies, Golden, Colorado 80401, United States; # PSI Center for Neutron and Muon Sciences, Paul Scherrer Institute, Forschungsstrasse 111, CH-5232 Villigen PSI, Switzerland; ∇ Laboratory for Neutron Scattering and Imaging, PSI Center for Neutron and Muon Sciences, Paul Scherrer Institute, Forschungsstrasse 111, 5232 Villigen PSI, Switzerland

## Abstract

Low-dimensional materials manifest structural anisotropy, quantum confinement, and tightly bound excitonic states, which make them attractive building blocks that can be assembled within three-dimensional laterally stitched heterostructures, stacked van der Waals solids, and complex moiré superlattices. Ion intercalation in the galleries between layered materials provides a means of modifying interlayer separation and coupling, but it is also known to drive the shearing of the layers. In this article, we explore the distinct ligand coordination environments afforded by vanadyl oxygens of singular [V_4_O_10_] sheets and examine how the size, polarizability, and stoichiometry of Group I cations sandwiched between such layers determine the interlocking of the sheets in stacked structures. Based on the topochemical insertion of alkali-metal ions into the layered λ-V_2_O_5_, we identify seven types of guest ion coordination sites discretized into four distinct regimes of interlayer shear in units of half octahedral widths. The coordination preferences of intercalated cations govern how they interlock 2D [V_4_O_10_] sheets and engender specific shear conformations. We present evidence that static and dynamic disorder in guest ion arrangement modulate the magnetic structure of the intercalated compounds based on electrostatic polarization, localization of charge and spin density, and lattice distortion. The results illustrate the use of topochemical ion insertion to modulate stacking relationships and magnetic transition characteristics.

## Graphical abstract

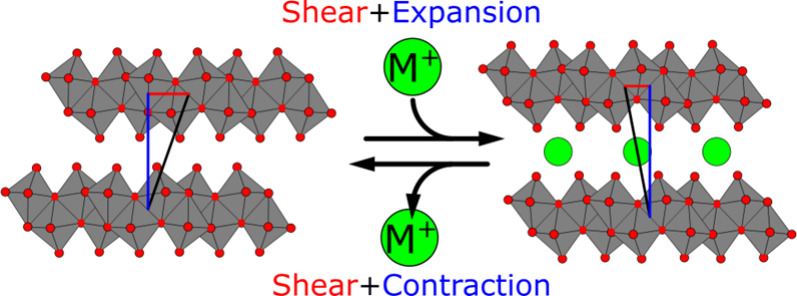

## Introduction

Dimensionally reduced materials exhibit electronic structure peculiarities and spin texturing arising from structural anisotropy, quantum confinement, and tightly bound excitonic states. In 2D materials, such spin and charge states manifest distinctive functionality such as topologically protected carrier transport, anomalous quantum Hall phenomena, valley degrees of freedom for spin-polarized transport, and in-plane ferroelectricity.^1–5^ Assembling similar or dissimilar 2D materials in configurations such as laterally stitched heterostructures, 3D layered stacks, and more complex twisted conformations provides access to a rich class of van der Waals solids with tunable interlayer coupling.^6–9^ Indeed, few-layered misaligned moiré superlattices of 2D materials manifest emergent properties stemming from collective electronic phases such as orbital magnetism, superconductivity, discrete quantum phases, and correlated insulator states.^10–12^

Notably, 2D materials can be stabilized in dimensionally reduced forms primarily because each layer is in itself coordinatively saturated and can thus be readily cleaved from 3D van der Waals solids. Aromatic residues and the anion sublattice of many layered materials provide a means of establishing Lewis acid–base interactions with surficial or intercalated cations.^13–15^ Ion insertion in galleries of layered materials is charge compensated by redox reactions with varying degrees of electron (de)­localization on the layered frameworks. Ion intercalation between 2D layers is key to a broad range of functionality. For example, the layered structure of many lithium-ion battery cathode materials (including the ubiquitous LiNi_*x*_Mn_*y*_Co_1–*x*–*y*_O_2_) affords rapid Li-ion diffusion and high energy density. Similarly, cations can be inserted without desolvation in galleries within layered materials to access intercalation pseudocapacitance charge storage mechanisms.^16,17^ Intercalated graphite adopts a variety of stacking arrangements depending on the identity and stoichiometry of the intercalant species. The resulting superlattice structures fold the original graphite Brillouin zone inward and induce new interactions between electronic states at overlapping *k*-points.^18^ Similar symmetry breaking by the removal of potassium from the kagome material K_1_V_6_Sb_6_ to form K_0.1_V_6_Sb_6_ lifts the topological protection of the insulating state and imparts a metallic band structure.^19,20^ In transition metal dichalcogenides (TMDCs), a well-studied class of layered intercalation hosts, the fundamental building block comprises three covalently bonded atomic X–M–X planes in each layer;^21^ the relatively high-symmetry configurations lead to a limited set of stacking sequences.^22,23^ For instance, MoS_2_ is a canonical example of a TMDC with rich intercalation chemistry. Insertion of guest ions into few-layered MoS_2_ can install and fine-tune new magnetic states,^24,25^ tune electrical conductivity by modulating electron-hopping transport,^26^ and even induce structure transitions within the layers of the host MoS_2_ lattice.^27,28^ MXenes, layered transition metal (M) carbides or nitrides (X), have also recently attracted attention. MXenes are prepared via topochemical methods that install pendant termination moieties facing interlayer interstices.^29^ The ability to select termination species during synthesis (commonly =O, −OH, −F, and −Cl) introduces an additional dimension of compositional and structural flexibility, with interlayer interactions and guest ion mobility being highly dependent on the density and identity of terminations.^30^

In contrast, layered oxides are much less systematically explored, and their lower symmetry and strong electron correlation yield a considerably greater diversity of stacking arrangements. Layered transition metal oxides exhibit a broad range of ion–lattice interactions, which we demonstrate here to stabilize complex shear relationships. As an exemplar, the five atomic layer structure and relatively few symmetry elements of V_2_O_5_ imbue rich but discretized shear relationships between proximate layers. The sensitivity of the electronic structure to ion insertion via compensatory vanadium reduction manifests strong charge and spin ordering, which makes these materials interesting “corrals” for defining electron and spin localization based on ion insertion chemistry. From a functional perspective, wide-bandgap transition metal oxides with polymorphism and a variety of structural, electronic, and magnetic structure transformations represent a rich counterpoint to semiconducting TMDCs and metallic MXenes.

Insertion of lithium into the thermodynamic layered α-V_2_O_5_ structure induces distortions and eventually the rearrangement of V–O bonds, accumulating in a series of structural phase transformations, which deleteriously impact its performance as a Li-ion battery cathode.^31^ These behaviors arise from strong coupling between electron transfer and (de)­localization, the evolution of band structure, and ensuing electron–phonon-coupling-mediated lattice distortion resulting from ion intercalation and the accommodation of guest ions in interstitial sites. A systematic understanding of the guest–host interactions that govern the accessibility, geometry, and occupancy of intercalant sites is crucial to developing design principles and a systematic taxonomy of 2D transition metal oxides that exhibit complex shear stacking patterns.^17,32,33^

The double-layered vanadium oxide bronzes are a valuable test-bed for isolating and studying how intercalants govern the structure and function of layered intercalated oxides. These materials and their analogues host a variety of monovalent and divalent pillaring cations, as well as water and even relatively large organic molecules, often across broad stoichiometric ranges.^34–36^ They are of additional interest due to their proclivity to manifest nonlinear dynamical current–voltage and resistance versus temperature characteristics critical to the design of electrothermal neurons.^37,38^ Pronounced nonlinearities such as temperature- and voltage-induced insulator–metal transitions have been demonstrated in δ-Tl_0.5_V_2_O_5_,^39^ δ-Na_0.5_V_2_O_5_,^40^ δ-Sr_0.5_V_2_O_5_,^40^ δ-Ag_*x*_V_2_O_5_,^41^ δ-K_0.5_V_2_O_5_,^42,43^ and ε-Cu_0.9_V_2_O_5_.^44^ The latter two materials display negative differential resistance across their resistivity transitions, in principle rendering them suitable as active materials in neuron-emulative devices. Despite the rich diversity of electronic transitions, little is known about the factors that relate guest ion identity, stoichiometry, and arrangement to the shear and stacking arrangement and resulting electronic structure. As such, this work expands the systematic understanding of ion intercalation in layered vanadium oxides akin to that established for TMDCs and MXenes and enables the connection of shear relationships and transformations to ion composition and stoichiometry, which represents a key elaboration of concepts of chemical pressure.

Famously, the xerogel V_2_O_5_ phase was identified by total scattering studies to comprise nanoconfined water molecules intercalated in 2D galleries between infinite [V_4_O_10_] slabs isostructural to double-layered vanadium oxide bronzes.^45^ The [V_4_O_10_] slab comprises zigzag chains of edge-shared VO_6_ octahedra extended along the *b*-direction, which corner-share along *a* to form sheets that in turn connect in pairs along *c* by additional corner-sharing. Unlike the single-layered edge-connected V_2_O_5_ sheets found in the α-V_2_O_5_ polymorph, which are prone to extensive puckering,^46^ rigid [V_4_O_10_] slabs retain their planar geometry in intercalated ternary structures. Pendant vanadyl moieties on each face of the slab delineate three distinct cavities where ions can be situated, designated in Figure 1B as T_1_ (triangular), T_2_ (triangular), and R (rectangular), whereas the rigidity of the [V_4_O_10_] slabs ensures that the shape and arrangement of these pockets are consistent across guest ion species.

**Figure 1:**
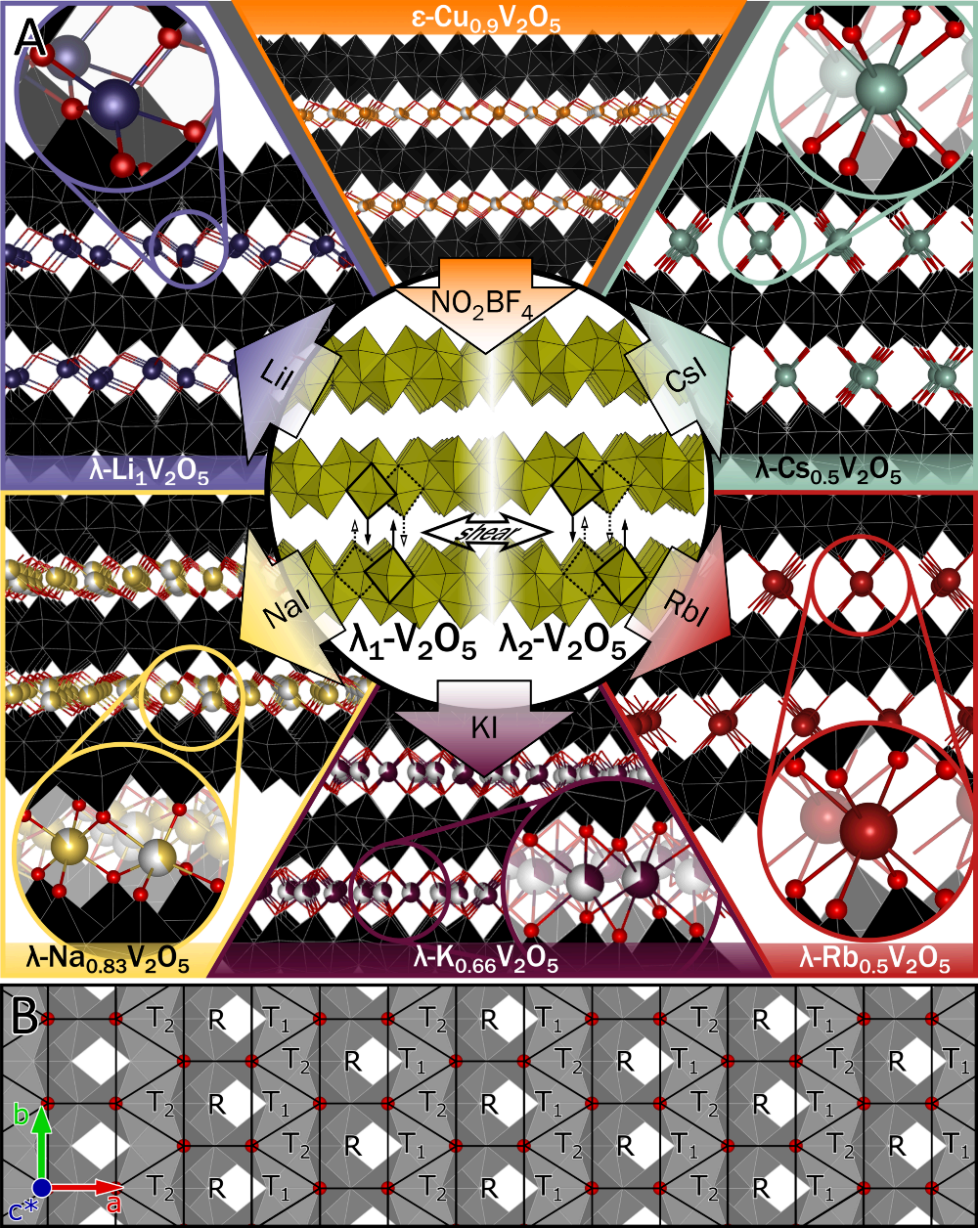
Topochemical ion insertion in double-layered V_2_O_5_. (A) Schematic depiction of topochemical intercalation of Group I ions between layers of λ-V_2_O_5_ and resulting λ-M_*x*_V_2_O_5_ structures. Insets show the coordination geometries of intercalated ions. VO_6_ octahedra are depicted in black, oxygen ions in red, copper ions in orange, lithium ions in indigo, sodium ions in yellow, potassium ions in maroon, rubidium ions in red, and cesium ions in teal. (B) Interstitial surface of a double-layered [V_4_O_10_] slab with coordination pockets labeled as R, T_1_, and T_2_.

In this article, we explore the distinct ligand coordination environments afforded by vanadyl oxygens of singular [V_4_O_10_] sheets and examine how intercalated monovalent cations sandwiched between such layers govern the stacking of the sheets in stacked structures. The size, stoichiometry, and hardness of the intercalated monovalent ions determine interlayer shear relationships, which are discretized in units of half octahedral widths. Intercalated ions sandwiched between two [V_4_O_10_] slabs can be coordinated in symmetric or asymmetric configurations corresponding to the same or different pockets on each side, which yield discrete shear relationships between the layers. Herein we seek to determine how the properties of guest ions—size, charge, polarizability/hardness, and outer-shell electron configuration—mediate their coordination preference and the layer geometry of the host material. Specifically, we use redox-mediated topochemical ion exchange to prepare M_*x*_V_2_O_5_ compounds, where M spans Group I cations. The chosen guest species, all alkali metals, are closed-shell monovalent ions, enabling ionic radius and polarizability to be mapped to layer sliding and electronic structure. X-ray diffraction analyses reveal a direct relationship between the radius of an intercalated ion and its coordination environment and host lattice shear structure. The electronic structures of the materials are examined using X-ray absorption near-edge structure (XANES) and extended structure (EXAFS) spectroscopy, hard X-ray photoelectron spectroscopy (HAXPES), and magnetic susceptibility experiments, which are further interpreted with the help of electron localization function (ELF) calculations via density functional theory (DFT).

## Results and Discussion

### Synthesis, Topochemical Conversion, and Electronic Structure

Figure 1A schematically depicts the synthesis of the λ-M_*x*_V_2_O_5_ materials discussed in this work. λ-V_2_O_5_ was prepared via the oxidative removal of copper from the planar interstices of ε-Cu_0.9_V_2_O_5_ using NO_2_BF_4_ according to eq 1:^47^

1 $${\varepsilon ‐\mathrm{Cu}}_{x}V_{2}O_{5}\left(s\right)+2x{\mathrm{NO}}_{2}{\mathrm{BF}}_{4}\left(\mathrm{acetonitrile}\right)\to \lambda {\mathrm{‐V}}_{2}O_{5}\left(s\right)+x\mathrm{Cu}{\left({\mathrm{BF}}_{4}\right)}_{2}\left(\mathrm{acetonitrile}\right)+2x{\mathrm{NO}}_{2}\left(g\right)$$

The topotactic nature of this reaction preserves the connectivity of the metastable V_2_O_5_ host structure; double-layered slabs in the product are separated by a van der Waals gap instead of being bridged by Cu^+^ ions. The λ-V_2_O_5_ product retains a high degree of crystallinity, indicated by the narrowness of reflections in Supporting Figure S1, despite the removal of interstitial ions and the rapid kinetics of the deintercalation reaction.

λ-V_2_O_5_ prepared by this reaction comprises separate crystalline polymorphs (labeled λ_1_-V_2_O_5_ and λ_2_-V_2_O_5_ in the central panel of Figure[Fig fig1]A) related by an interlayer shear along the *a* crystallographic axis, but with nearly identical *a* and *b* lattice parameters and interlayer spacing.^47,48^ Only the structure of λ_1_-V_2_O_5_ has so far been reported from single-crystal X-ray diffraction.^47^ Supporting Figure S2 and Tables S1 and S2 show a λ_2_-V_2_O_5_ structure obtained from Rietveld refinement of synchrotron powder XRD data.^49^

The alkali-metal ions M = {Li, Na, K, Rb, Cs} were inserted into λ-V_2_O_5_ according to eq 2 to obtain the corresponding λ-M_*x*_V_2_O_5_ materials depicted in the side panels of Figure 1A:

2 $$\lambda {\mathrm{‐V}}_{2}O_{5}\left(s\right)+2xMI\left(\mathrm{acetonitrile}\right)\to M_{2x}V_{2}O_{5}\left(s\right)+xI_{2}\left(\mathrm{acetonitrile}\right)$$

The van der Waals gap between infinite [V_4_O_10_] slabs can accommodate a wide range of ionic radii, from 0.59 Å for Li^+^ to 1.74 Å for Cs^+^.^50^

Structures were solved based on Rietveld refinements to powder X-ray diffraction (PXRD) patterns, as shown in Supporting Figures S1–S7 and Tables S1–S8. Two entirely new compounds, λ-Na_0.83_V_2_O_5_ and λ-Cs_0.5_V_2_O_5_, have been stabilized by this topochemical method. A full solution for the previously inferred structure of λ-K_0.66_V_2_O_5_ ^51,52^ has been determined. A refinement of the λ-Na_0.83_V_2_O_5_ powder XRD pattern is shown in Figure 2A as an example, with the structure model showing the positions of Na^+^ ions between V_2_O_5_ layers coordinated to vanadyl oxygens. The 001 reflection of λ-Na_0.83_V_2_O_5_ at ca. *Q* = 0.72 Å^–1^, which corresponds to the distance between planes bisecting V_2_O_5_ layers perpendicular to the *c*-direction, is compared to those of the other intercalated species in Figure 2B. A clear monotonic correlation between the ion radius and layer expansion is observed that will be discussed further below.

**Figure 2:**
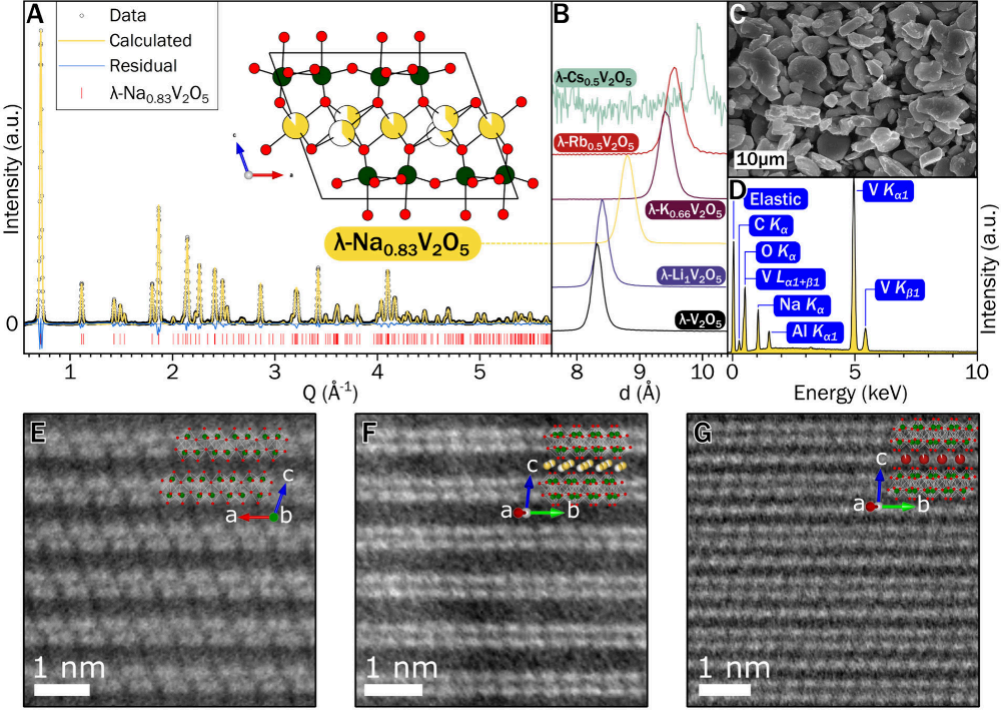
Structural characterization of double-layered λ-V_2_O_5_ and topochemically intercalated products. (A) Rietveld refinement of λ-Na_0.83_V_2_O_5_ from synchrotron powder XRD data; the refined structure is shown in the inset. (B) *d*-spacings of 001 reflections of λ-M_*x*_V_2_O_5_ materials corresponding to the layer spacing Σ for Group I topochemically intercalated ions from Li to Cs. (C) Scanning electron micrograph of λ-Na_0.83_V_2_O_5_ particles. (D) EDX spectrum of λ-Na_0.83_V_2_O_5_. (E–G) High-resolution scanning transmission electron micrographs of (E) λ_2_-V_2_O_5_, (F) λ-Na_0.83_V_2_O_5_, and (G) λ-Rb_0.5_V_2_O_5_, overlaid with Rietveld-refined crystal structures (green: vanadium; red: oxygen; yellow: sodium; maroon: rubidium).

Figure 2C shows the irregular platelet morphology inherited from the ε-Cu_0.9_V_2_O_5_ precursor, which corroborates the topotactic nature of the transformation. The corresponding energy-dispersive X-ray (EDX) spectrum in Figure 2D corroborates the complete replacement of Cu^+^ with Na^+^. The elemental compositions determined from Rietveld refinement, ICP/MS, and EDX for all materials are provided in Supporting Table S9 and are in good agreement. Note that the stoichiometries of λ-Na_0.83_V_2_O_5_ and λ-K_0.66_V_2_O_5_ indicate an incomplete occupancy of guest ion sites; the proximity of these values to small whole-number ratios (0.83 ≈ 5/6; 0.66 ≈ 2/3) is indicative of guest ion occupancy ordering (*vide infra*). We will hence refer to each compound by its stoichiometry, assuming either complete guest-site occupancy (for M = Li, Rb, Cs) or inferred whole-number-ratio occupancy ordering (M = Na, K). EDX composition maps for λ-Na_0.83_V_2_O_5_, λ-K_0.66_V_2_O_5_, λ-Rb_0.5_V_2_O_5_, and λ-Cs_0.5_V_2_O_5_ (Supporting Figure S8) demonstrate a homogeneous intercalant concentration between particles, which is further consistent with the occupancies inferred from Rietveld refinements (significant deviations in intercalant concentrations manifest considerable broadening of powder XRD reflections not observed here). Scanning transmission electron micrographs of λ_2_-V_2_O_5_, λ-Na_0.83_V_2_O_5_, and λ-Rb_0.5_V_2_O_5_ (Figure 2E–G, respectively) illustrate the layered structure of the V_2_O_5_ host, with V atoms arrayed in double-layered V_4_O_10_ units. Rb^+^ ions are observed in the interlayer space in Figure 2G.

The topochemically intercalated M_*x*_V_2_O_5_ compounds have been further characterized by V K-edge XANES, V L_3_- and O K-edge XANES, and core and valence band HAXPES measurements, which were acquired as described in the Experimental Procedures (Figure 3). Figure 4 illustrates the local coordination environments of Group I cations and the discretized shear relationships resulting from the specific coordination pockets available on each [V_4_O_10_] slab.

**Figure 3:**
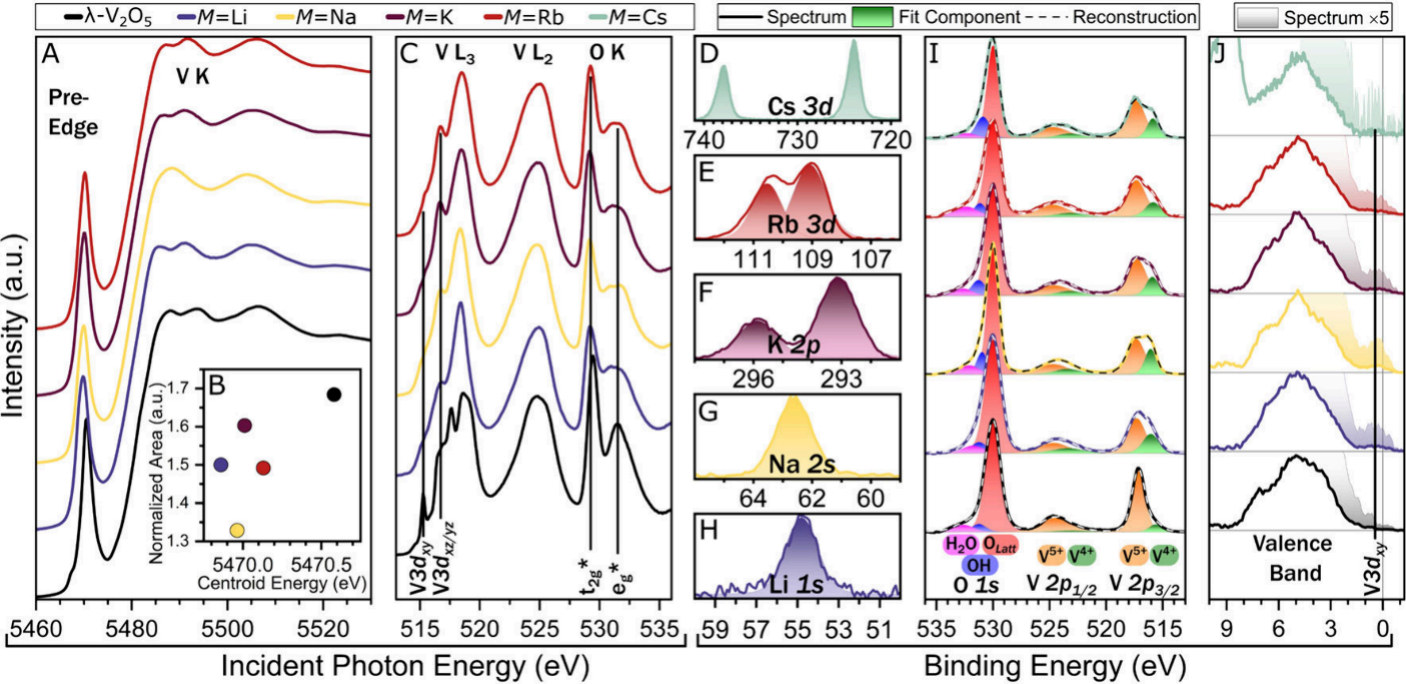
Electronic structure characterization of topochemically prepared M_*x*_V_2_O_5_. (A) V K-edge XANES spectra; (B) pre-edge peak area versus centroid energy derived from V K-edge XANES; and (C) V L_II,III_-edge and O K-edge XANES spectra contrasting λ-V_2_O_5_ and topochemically intercalated M_*x*_V_2_O_5_ compounds. (D–H) Core-level HAXPES spectra acquired at intercalated ion core levels; (I) O 1s and V 2p core-level HAXPES spectra (where the emergence of V^4+^ features evidence vanadium reduction upon ion intercalation); and (J) valence band HAXPES spectra, exhibiting distinct V 3d_*xy*_ features immediately below the Fermi level. All HAXPES spectra were acquired at 2 keV excitation.

**Figure 4:**
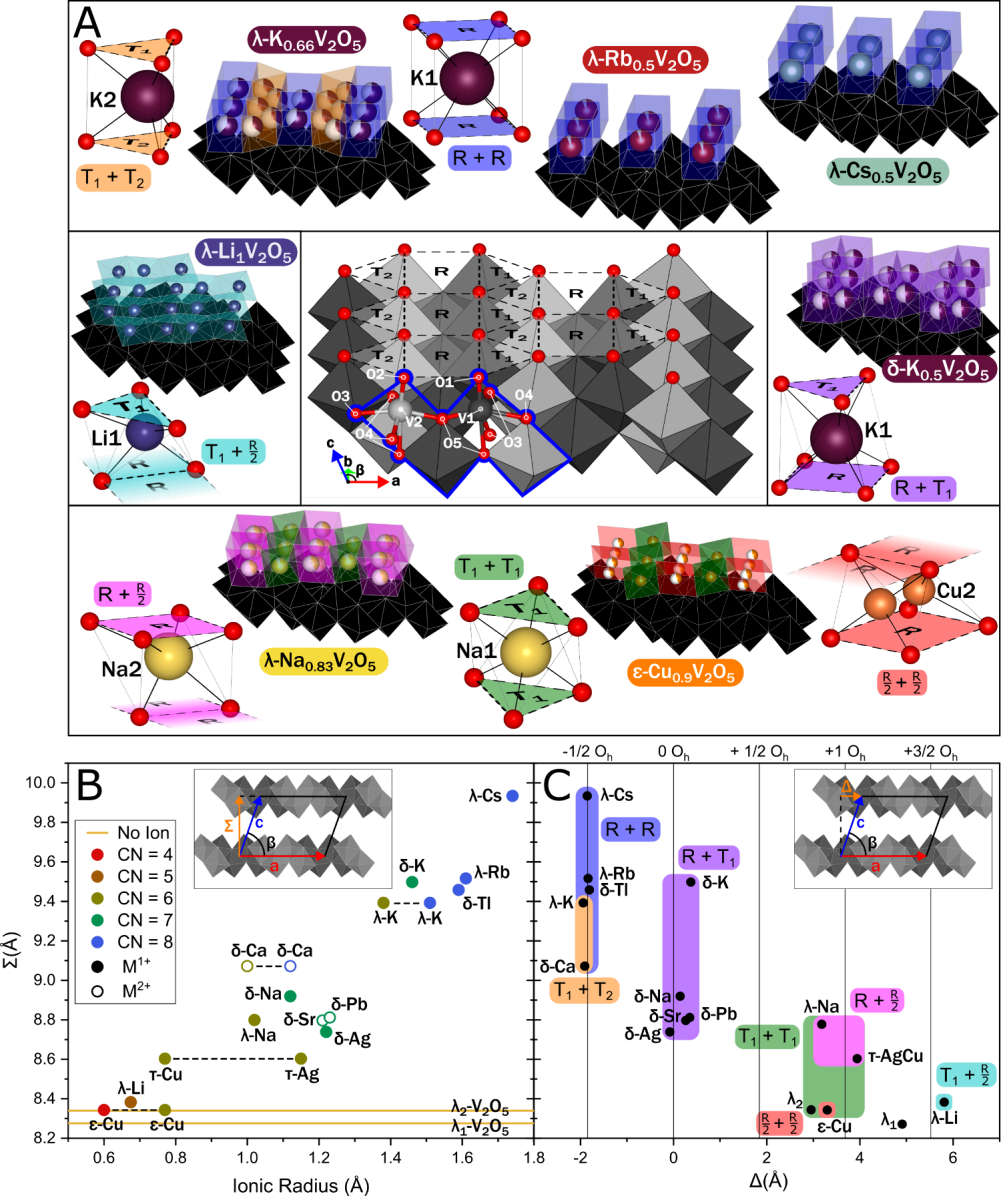
Coordination environments of intercalated cations in M_*x*_V_2_O_5_. (A) (Center) Arrangement of R, T_1_, and T_2_ half-sites defined by vanadyl oxygens across the interstitial surface of a V_4_O_10_ slab. The cutaway shows local coordination around V1 (dark gray) and V2 (light gray) sites, with the V_4_O_18_ unit outlined in blue. (Periphery) Interlayer arrangement of intercalated ions with local coordination environments annotated with half-site identities. For instance, T_1_ + T_1_ adopted by Na1 in λ-Na_0.83_V_2_O_5_ implies that Na coordinates to T_1_ on the lower [V_4_O_10_] slab and its image under two-fold rotation on the upper slab. R/2 + R/2 adopted by Cu2 in ε-Cu_0.9_V_2_O_5_ implies that Cu coordinates to a pair of oxygens making up one side of R on each slab. (B) Interlayer distance Σ versus intercalant ionic radius. Ions in different sites in the same structure are connected by dashed lines. Inset depicts the geometric definition of Σ. (C) Σ versus interlayer shear Δ, with data points highlighted by site types occupied in each material. Vertical lines indicate half-intervals of the VO_6_ octahedral width along the *a*-axis (O_h_). Inset depicts the geometric definition of Δ. Data from refs 36, 39, 47, and 62–67 as tabulated in Supporting Table S10.

We first examined the electronic structure implications of intercalating Group I cations between [V_4_O_10_] slabs. In addition to the white-line absorption edge at 5485 eV, the vanadium K-edge XANES spectra acquired for λ-V_2_O_5_ and λ-M_*x*_V_2_O_5_ plotted in Figure 3A feature a pre-edge absorption peak at ca. 5470 eV. This lower-energy feature arises from formally forbidden V 1s → V 3d dipole transitions enabled by the displacement of vanadium ions from octahedral centers, which breaks orbital symmetry and induces V 3d–4p hybridization.^53,54^ The area and energy position of this peak are thus sensitive probes of vanadium coordination and oxidation states, respectively, and are plotted in Figure 3B. The centroid energy decreases with increasing concentration of intercalated ions (concomitant with a greater reduction of pentavalent vanadium to tetravalent vanadium).

Figure 3C plots V L_II_- and L_III_-edge and O K-edge XANES spectra. Features at the V L_III_-edge represent V 2p → V 3d transitions, with fine structure arising from the crystal field splitting of empty V 3d orbitals.^55,56^ The suppression of the V 3d_*xy*_ feature can be ascribed to Pauli blocking and is consistent with vanadium reduction upon ion intercalation.^56,57^ The 3d_*xz*/*yz*_ feature originally split in λ-V_2_O_5_ coalesces into a single resonance in λ-M_*x*_V_2_O_5_, corroborating the decreased V off-centering interpreted from V K-edge spectra. While the V L_II_-edge features arise from similar transitions to those at the L_3_-edge, they are broadened by Coster–Kronig Auger emission processes.^56,57^

The O K-edge transitions from O 1s to O 2p states in Figure 3C serve as a measure of O 2p hybridization with empty V 3d t_2g_ and e_g_ states.^53,56^ The variation of V−O covalency within the layered frameworks is observed as a decrease in t_2g_/e_g_ * ratio with higher intercalant concentration. Element-specific X-ray absorption spectroscopy measurements thus corroborate that charge compensation of intercalated ions derives from vanadium reduction; the coupling of localized electrons on the [V_4_O_10_] slabs with the intercalated ion mediated through vanadyl moieties gives rise to polaronic character given the large bandgaps of double-layered λ-V_2_O_5_ polymorphs (*vide infra*).^53,58^

The intercalant core-level HAXPES spectra in Figure 3D–H confirm the successful ion insertion. The Gaussian fits to lineshapes are consistent with a single oxidation state for each intercalant. The intensity ratios of V^4+^ versus V^5+^ features in the V 2p_3/2_ core-level HAXPES spectra in Figure 3I are directly correlated with intercalant concentration and corroborate that vanadium reduction accompanies ion insertion as observed in the XANES spectra. Figure 3J compares the valence band spectra of λ-V_2_O_5_ and λ-M_*x*_V_2_O_5_. The majority of the valence band lies below a binding energy of 2 eV and is derived from O 2p states hybridized with V 3d states, whereas V 3d_*xy*_ states visible at 0–2 eV span the Fermi level and are suggestive of the stabilization of small polarons, which are further discussed below in the context of electron localization function (ELF) maps (*vide infra*).^59^

### Coordination Preferences and Discretized Shear Relationships

In the double-layered vanadium oxide bronzes, the V_2_O_5_ substructure consists of slabs constructed by edge-sharing of VO_6_ octahedra. The electronegativity of formally V^5+^ ions and the resultant V–O covalency induce an off-centering of the vanadium ion to form short V1=O1 and V2=O2 vanadyl bonds^49,60^ oriented toward the interlayer ions (Figure 4A, center). These vanadyl oxygens define a two-dimensional lattice of triangular (T_1_ and T_2_) and rectangular (R) polygons (Figure 1B). Examination of the double-layered bronzes studied here and their structural analogues mined from the literature reveals that the local coordination environment of the intercalated ion is formed by the association of one polygon (or fraction thereof) from the lower polygonal lattice with another from its inverted upper counterpart. Seven such combinations are observed among the structures, which are named here for the vanadyl plane polygons from which they are formed. Examples of each, with accompanying parent structures, are shown in the side panels of Figure 4A.^52^ The precursor material ε-Cu_0.9_V_2_O_5_ ^44,61^ intercalates Cu^+^ ions in two sites: Cu1 coordinates to one triangular T_1_ polygon on each layer whose antiprismatic orientation creates an octahedral T_1_ + T_1_ site, whereas Cu2 coordinates to one pair of oxygens from each of two R polygons to form a (distorted) square-planar R/2 + R/2 site.

Treating λ-V_2_O_5_ with LiI yields λ-Li_1_V_2_O_5_ (Supporting Figure S3 and Table S3), which is well matched to the structure determined from a single crystal prepared using the more powerful reducing agent *n*-butyllithium.^47^ Li^+^ ions coordinate to a T_1_ polygon in one layer and to one side of an R polygon in the other, thereby forming a five-coordinated trigonal bipyramidal T_1_ + R/2 local coordination environment. Notably, this is not one of the Li-site geometries anticipated by Galy in prior analyses,^34^ which remain to be observed.

The novel phase λ-Na_0.83_V_2_O_5_ is nearly isostructural to ε-Cu_0.9_V_2_O_5_ (Figure 2 and Supporting Figure S4 and Table S4);^61^ both intercalated compounds host roughly half of their intercalated ions (Na1 and Cu1, respectively) in chains of six-coordinated distorted octahedral T_1_ + T_1_ sites. The remaining intercalated cations reside in parallel chains of interstitial sites (Na2 in six-coordinated trigonal prismatic R + R/2 and Cu2 in four-coordinated distorted square planar R/2 + R/2) with twice the multiplicity but roughly half the site occupancy. The latter occupancies are explicable by the proximity of these sites along the *b*-direction, which forbids the simultaneous occupation of adjacent sites and suggests the possibility of short-range occupancy-defined ordering.

λ-K_0.66_V_2_O_5_ (Supporting Figure S5 and Table S5), first identified by Pouchard and Hagenmuller,^51^ is essentially identical to the ν-K_0.6_V_2_O_5_ structure inferred by Galy^34^ from the solved structure of Ca_0.6_V_2_O_5_.^65^ Eight-coordinated rectangular prismatic R + R sites host K1 ions at ca. 2/3 occupancy and are each flanked by two trigonal prismatic T_1_ + T_2_ K2 sites at ca. 1/3 occupancy. As in the sodiated structure, site proximities and fractional occupancies strongly suggest local occupancy ordering. λ-Rb_0.5_V_2_O_5_ (Supporting Figure S6 and Table S6) is identical to the ν-Rb_0.5_V_2_O_5_ previously reported from a single-crystal diffraction study^36^ and exhibits the same R + R intercalation site geometry as the potassium analogue, albeit fully occupied and unaccompanied by the filling of neighboring T_1_ + T_2_ sites. Finally, the novel compound λ-Cs_0.5_V_2_O_5_ (Supporting Figure S7 and Tables S7 and S8) is isostructural to λ-Rb_0.5_V_2_O_5_ apart from an increased layer spacing. Indeed, the additional interlayer void volume created by this expansion exacerbates the propensity of double-layered bronzes to intercalate atmospheric water,^45^ which renders magnetic susceptibility measurements more challenging. Supporting Figure S9 depicts *in situ* powder X-ray diffraction patterns of a hydrated λ-Cs_0.5_V_2_O_5_ specimen heated from 25 to 300 °C. Heating the hydrated specimen to 75 °C for approximately 3 h recovers the original λ-Cs_0.5_V_2_O_5_ structure.

To facilitate further structure comparisons, we note that across the entire series of Group I intercalated M_*x*_V_2_O_5_ bronzes, only two unit cell parameters (*c* and β) can be varied without distorting the vanadium oxide substructure or disrupting crystal symmetry (see Supporting Figure S10 and the related discussion). The layer stacking geometry of the double-layered bronzes can thus be defined solely based on two degrees of freedom, the interlayer shear along [100] and interlayer separation along [001]. We define these respectively as Δ^34^ and Σ, according to eqs 3 and 4 (and shown in the insets in Figure 4B,C):

3 Δ≡c×cos(β)

4 Σ≡c×sin(β)

where *c* and β denote the *c* unit cell length and the monoclinic unique axis angle, respectively. The crystallographic parameters of a variety of double-layered bronzes reported in literature sources^62–68^ are tabulated in Supporting Table S10, along with details of their idealization.

The plot of interlayer spacing Σ versus ionic radius^50^ shown in Figure 4B corroborates their monotonic correlation. Divalent ions do not significantly deviate from the trend exhibited by their monovalent counterparts. Intercalated ions are also clustered according to coordination number, as expected from radius ratio considerations.^69^

The plot of Σ versus Δ in Figure 4C encapsulates the relationship between the identity of intercalated ions, their local coordination environment, and the shear relationships they engender between the V_2_O_5_ layers. The periodicity in Δ with which vanadyl lattice polygons overlap to form viable intercalation sites leads the clustering of structures in narrow bands in Δ, roughly corresponding to half-increments of the VO_6_ octahedral width along *a*.^34^ The apparent Σ–Δ regions of stability are highlighted for each coordination site as defined by pockets defined on upper and lower slabs (Figure 1). Several sites show a dependence on Σ, as well as Δ. T_1_ + T_2_ sites are not found above Σ = 9.4 Å, as the small triangular polygons provide insufficient solid angle coverage to adequately stabilize the positive charge of larger ions. The R/2 + R/2 site is similarly inappropriate for all but the smallest ions such as four-coordinated Cu^+^. Neglecting V_2_O_5_ substructure distortions, Σ and Δ together entirely encapsulate the dimensions and geometry of coordination sites. As such, intercalating ions close to one another on this plot will readily form solid solutions, whereas those more distant are likely to enable site-selective modification on distinct interstitial sites.^70,71^

The powder XRD patterns of λ-Na_0.83_V_2_O_5_ and λ-K_0.66_V_2_O_5_ (Supporting Figures S4 and S5) contain several reflections that can be indexed as modulation satellites with modulation vectors *q* = (0, 1/3, 0) and *q* = (0, 1/6, 0), respectively (Supporting Figure S11). The near-commensurability of Na1 (0.883 ≈ 5/6) and Na2 (0.392 ≈ 5/12) site occupancies in λ-Na_0.83_V_2_O_5_ and K1 (0.631 ≈ 2/3) and K2 (0.351 ≈ 1/3) site occupancies in λ-K_0.66_V_2_O_5_ suggests that these may arise from ordered site occupancy, although their low intensity suggests that the ordering is relatively short-range. Similar structure modulation has been observed in single crystals of ε-Cu_0.9_V_2_O_5_ coincident with an insulator–metal transition underpinned by correlated order–disorder of Cu ions.^44^ By contrast, Li, Rb, and Cs site occupancies in the corresponding structures are approximately 1, which does not offer such a possibility of guest ion ordering.

Stoichiometry adds further nuance to the plot in Figure 4C. Several ions span multiple structure regimes, differing only in stoichiometry (i.e., Na in δ-Na_0.5_V_2_O_5_ and λ-Na_0.83_V_2_O_5_, K in δ-K_0.5_V_2_O_5_ and λ-K_0.66_V_2_O_5_). The R+T_1_ sites in the δ-phase almost completely fill the interlayer space but do so inefficiently from a capacity standpoint: one such site is available per V_2_O_5_ formula unit, but their proximity limits them to half occupancy as reflected in the overall stoichiometry of δ-M_0.5_V_2_O_5_. The R + T_1_ site in the δ-M_0.5_V_2_O_5_ structure family has been described as a hybrid of the R + R and T_1_ + T_2_ sites of the λ-K_0.66_V_2_O_5_ structure adopted at higher potassium concentrations.^34^ By similar reasoning, it can be related to the T_1_ + T_1_ and R + R/2 in λ-Na_0.83_V_2_O_5_. The shear transformation aligns the T_1_ pocket in the R + T_1_ site with that of its neighbor to generate an octahedral T_1_ + T_1_ site, and aligns an R pocket with the shared R–R edge of its opposite neighbor to generate a trigonal prismatic R + R/2 site. Shear transformations for both materials are depicted in Supporting Figure S12. These results suggest that electrochemical and topotactic insertion and removal of ions with sufficient chemical potential can induce synchronized in-plane cation reordering and drive structure transitions between the high- and low-stoichiometry structure regimes. Indeed, the specific site preferences and their preferred shear relationships appear key to deciphering the sliding behavior observed in layered Na-ion cathodes.^72,73^

The extensive diversity of interstitial sites occupied by guest ions based on stacking configurations of the double-layered bronzes warrants comparison to those observed in intercalated TMDCs and MXenes. The predominant feature of the double-layered bronzes is the low symmetry of the [V_4_O_10_] slabs (particularly in the arrangement of O^2–^ moieties, which coordinate to guest ions) compared to their counterparts in TMDCs and MXenes. The higher-symmetry hexagonal structures of chalcogenide planes in TMDCs and (filled) termination planes in MXenes define only triangular pockets, equivalent to T_1_ and T_2_ pockets in the double-layered bronzes, and thus provide access to fewer guest ion coordination geometries.^22,74^ Analogous to T_1_ + T_1_ sites in λ-Na_0.83_V_2_O_5_, Na ions in several TMDCs adopt octahedral coordination but only at high stoichiometries. Larger alkali-metal ions (and Na at low stoichiometries) occupy instead vertical triangular pyramidal sites equivalent to the T_1_ + T_2_ sites observed in λ-K_0.66_V_2_O_5_ and δ-Ca_0.6_V_2_O_5_ (although in TMDCs, the two triangular pockets involved are symmetry-equivalent). As such, most of the coordination geometries presented in Figure 4 that involve the R pocket are not equivalent among intercalated TMDCs. Similarly, the preference of MXenes for octahedral versus trigonal stacking is dependent on the composition of their termination layers,^75^ and this strongly affects the migration of guest ions.^30^ The preferences of particular ions for octahedral or trigonal coordination similarly depend on the composition and structure of the termination planes.^30,76^ In contrast, owing to their lower symmetry, our systems under consideration enable clean isolation of distinctive and discretized shear relationships: the connection of shear transformations to ion composition and stoichiometry provides a means of finely modulating electron and spin localization through tuning of shear relationships. As another key distinction, double-layered vanadium oxide bronzes also frequently situate guest ions in two sites simultaneously, as in the case of λ-Na_0.83_V_2_O_5_, λ-K_0.66_V_2_O_5_, δ-Ca_0.6_V_2_O_5_, ε-Cu_0.9_V_2_O_5_, and τ-Ag_*x*_Cu_*y*_V_2_O_5_. These materials hold promise for studying ordering effects resulting from complex intralayer motion and their interaction with localized charge on the host lattice.

To further examine the structure preferences of λ-M_*x*_V_2_O_5_ materials, we have performed shear and exfoliation energy calculations, as described in the Experimental Procedures. Figure 5A depicts the calculated energy for shear relationships in the vicinity of the measured shear values for material compositions approximating those determined experimentally (solid lines), as well as for a uniform M_0.5_V_2_O_5_ composition to facilitate comparison between ions. No consistent trend emerges with respect to guest ion atomic number (or, therefore, radius) or concentration, which leads us to conclude that differences arise primarily from the rigidity of the particular coordination geometry of each ion against shearing distortion. This is supported by the similarities observed for three isostructural materials with the same concentration (λ-Rb_0.5_V_2_O_5_, λ-Cs_0.5_V_2_O_5_, and λ-K_0.5_V_2_O_5_). Intriguingly, λ-K_0.75_V_2_O_5_ with both T_1_ + T_2_ and R + R sites occupied is much more easily sheared than λ-K_0.5_V_2_O_5_ with only R + R sites occupied, suggesting that the distorted cubic R + R site is destabilized by filling the adjacent T_1_ + T_2_ sites. λ-Li_*x*_V_2_O_5_ and λ-Na_*x*_V_2_O_5_ show asymmetric energy profiles, with positive shear values significantly less stable than negative ones, especially for the reduced *x* = 0.5 stoichiometries. These results suggest the presence of adjacent shear regimes with viable coordination sites but with multiplicity too low to host the simulated guest ion concentrations.

**Figure 5:**
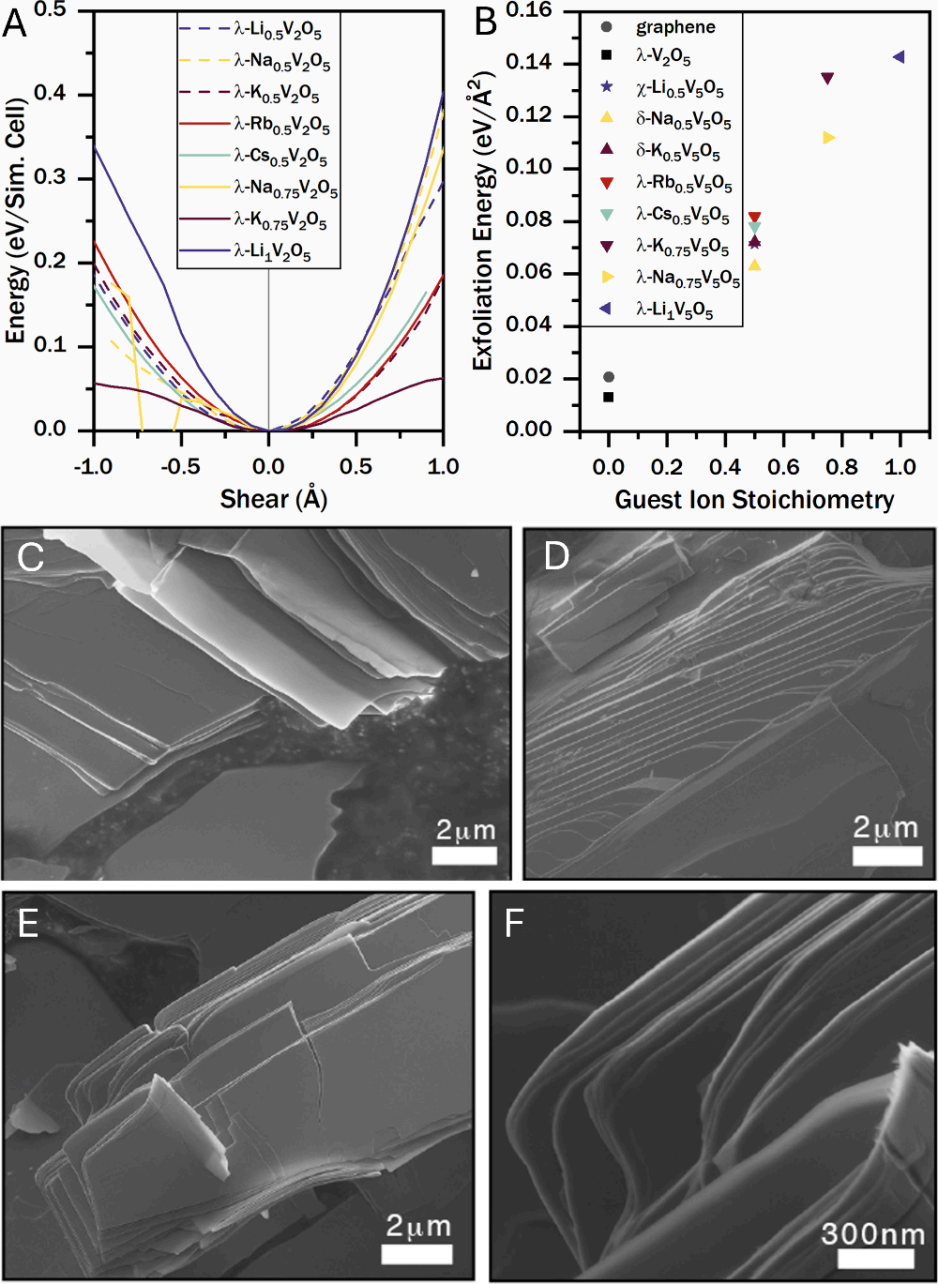
Shear and exfoliation of double-layered bronzes. (A) Calculated energy versus shear relationships. Solid lines approximate experimentally determined compositions, whereas dashed lines were calculated with a uniform M_0.5_V_2_O_5_ stoichiometry to facilitate comparison between ions. (B) Exfoliation energies for double-layered bronzes, including λ-M_*x*_V_2_O_5_ materials discussed in the main text (symbols), with graphene as a reference. Interlayer attraction in λ-V_2_O_5_ is weak, which renders these materials readily exfoliatable. In intercalated samples, the exfoliation energy increases roughly in proportion to guest ion concentration. Note that stoichiometries have been adjusted from measured values to facilitate calculations. (D–F) Scanning electron microscopy images of tape-exfoliated (C, D) λ-V_2_O_5_ and (E, F) λ-K_0.66_V_2_O_5_, showing flakes with thicknesses of tens of nanometers and step-edges from mechanical layer shearing.

The quasi-2D nature of λ-M_*x*_V_2_O_5_ suggests that exfoliation to few-layered thicknesses may be a viable method for tuning material properties, as has been demonstrated for other layered materials like graphene^77^ and TMDCs.^78,79^ To assess the feasibility of such studies in λ-M_*x*_V_2_O_5_, we performed exfoliation energy calculations and preliminary mechanical exfoliation studies. Calculations (Figure 5B) predict that the deintercalated host λ-V_2_O_5_ is especially facile to exfoliate with only half the exfoliation energy of graphene, evidenced by the visible flakes in Figure 5C,D. Ion insertion increases the exfoliation energy roughly in proportion to the guest ion stoichiometry, which implies that guest ions contribute to layer adhesion via coordination to vanadyl oxygen moieties. Layer separation and flake formation are readily accessible in tape-exfoliated λ-K_0.66_V_2_O_5_. Figure 5E,F indicates that the ion-intercalated materials remain exfoliatable.

### Functional Implications of Ion Intercalation and Shear Relationships

The temperature dependence of the magnetic susceptibility, χ­(T), of λ-V_2_O_5_, λ-Li_1_V_2_O_5_, λ-Na_0.83_V_2_O_5_, λ-K_0.66_V_2_O_5_, λ-Rb_0.5_V_2_O_5_, and λ-Cs_0.5_V_2_O_5_ were measured under zero-field-cooled (ZFC)–field-cooled (FC) conditions in the temperature range of 2–400 K under an external field of 0.1 T as shown in Figure 6A,B and Supporting Figures S13 and S14. The FC susceptibility of the parent λ-V_2_O_5_ compound is characteristic of weak paramagnetism, which is consistent with the formally pentavalent 3d^0^ electron configuration of vanadium. The FC susceptibility of several of the topochemically intercalated compounds reveals relatively broad transitions (Figure 6A) at λ-Li_1_V_2_O_5_ (∼89 K), λ-K_0.66_V_2_O_5_ (∼122 K), λ-Rb_0.5_V_2_O_5_ (∼168 K), and λ-Cs_0.5_V_2_O_5_ (∼172 K). The magnetic susceptibility near the transition temperature generally tracks with guest ion concentration, which indicates that spins are localized on reduced vanadium centers within the [V_4_O_10_] slabs, compensating for the charge of the intercalated monovalent Group I ions.

**Figure 6:**
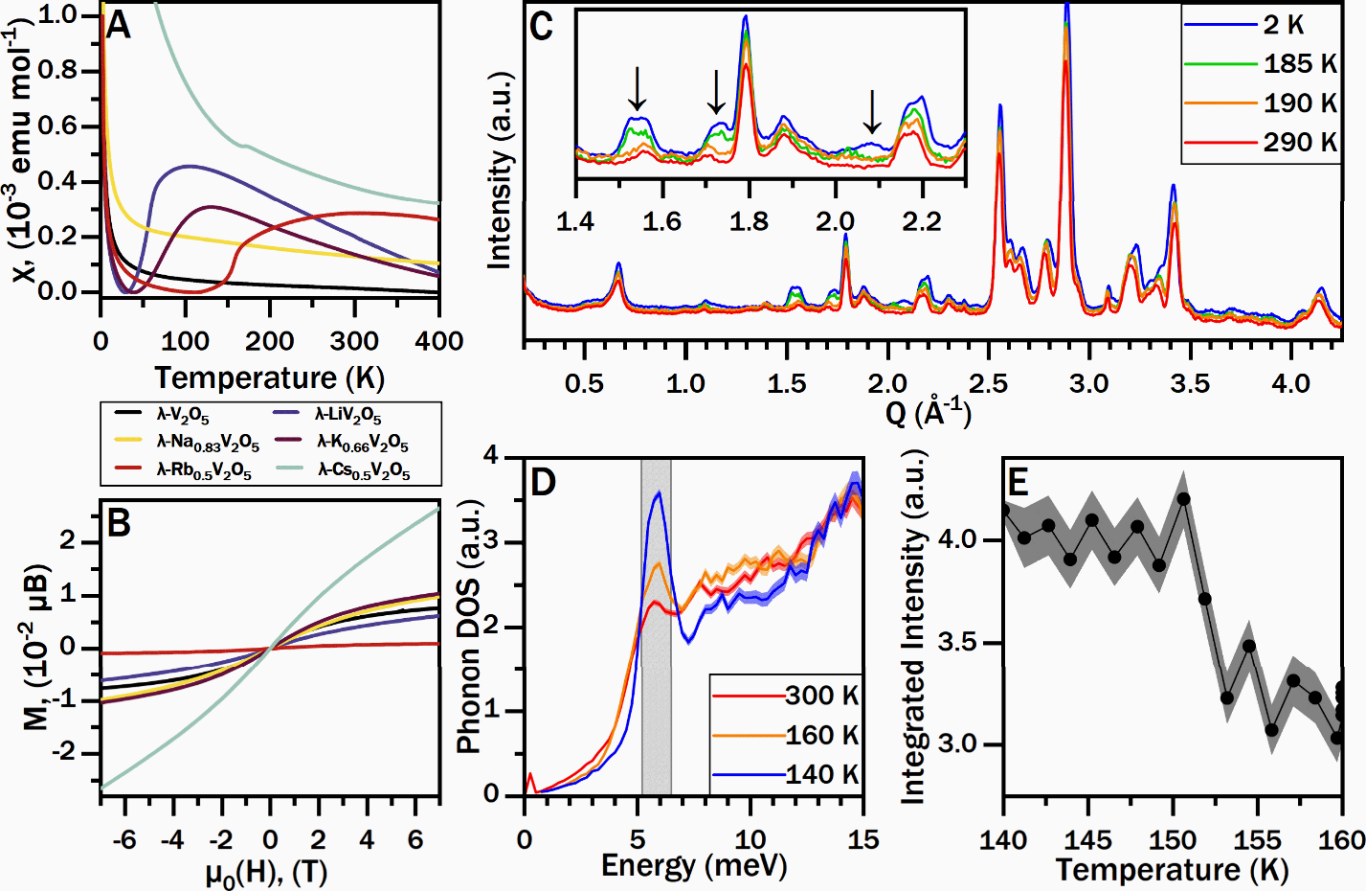
Magnetometry and neutron scattering measurements. (A) Field-cooled temperature-dependent magnetic susceptibility and (B) field-dependent magnetization (at 2 K) curves for λ-V_2_O_5_ and λ-M_*x*_V_2_O_5_ specimens. (C) Temperature-dependent neutron diffraction patterns for λ-K_0.66_V_2_O_5_, in which three new (magnetic) reflections appear at low temperatures, magnified in the inset. (D) Temperature-dependent phonon density of states for ε-Cu_0.9_V_2_O_5_ extracted from inelastic neutron scattering data. (E) Intensity of the 6 meV phonon peak, integrated over the gray region in (D), showing a steep drop at the ion-ordering transition temperature at ca. 150 K.

Figure 6B illustrates the field-dependent magnetization (*M* versus *H*) measurement at 2 K under an applied magnetic field ranging from −7 to +7 T. The magnetization curve of all the compounds shows an initial rapid rise at low fields followed by a linear increase up to 7 T without saturation. The absence of saturation up to 7 T indicates a tendency toward antiferromagnetic (AFM) interactions between V atoms. Brillouin-function fits to the *M*–*H* data (Supporting Figure S15) yield saturation magnetization (*M*_sat_) values of 2.4 × 10^–3^–6.0 × 10^–2^ μ_B_ for the intercalated compounds, which are lower than the expected value of 1.0 μ_B_ (*M*_sat_ = *ngS* μ_B_) per formula unit for a localized V^*n*+^ (3d^1^, *S* = 1/2) configuration, indicating that not all moments contribute fully to the field-induced magnetization.^80,81^ The curves also display a sigmoidal shape, characteristic of weak ferromagnetic (FM) interactions along the exchange interaction pathway. This complex magnetic behavior observed in *M*(*H*) can be ascribed to a competing AFM state with an intrinsic weak FM state (*vide infra*).

The observed upturns in susceptibility at 28, 40, 125, and 165 K in λ-Li_1_V_2_O_5_, λ-K_0.66_V_2_O_5_, λ-Rb_0.5_V_2_O_5_, and λ-Cs_0.5_V_2_O_5_ likely stem from this ferromagnetic exchange coupling, possibly originating from a canted-spin arrangement of V^*n*+^ centers or from isolated paramagnetic spins or paramagnetic impurities.^66,82^ The temperature at which this upturn occurs increases with decreasing guest ion concentration. All the compounds exhibit paramagnetic behavior above the transition temperatures, which follows a modified Curie–Weiss dependence for paramagnetic spins (Supporting Figure S16 displays the respective fits):

5 $$\chi =\chi_{0}+C/\left(T-\theta \right)$$

where θ denotes the Curie–Weiss temperature and *C* is the Curie constant. The effective magnetic moments μ_eff_ obtained for the V^*n*+^ ions are 1.71, 1.73, 1.72, and 1.71 μ_B_ for λ-Li_1_V_2_O_5_, λ-Na_0.83_V_2_O_5_, λ-K_0.66_V_2_O_5_, and λ-Cs_0.5_V_2_O_5_ respectively. These values are consistent with the expected effective moments of V^4+^ ions (*S* = 1/2 ; μ_eff_ = 1.73 μ_B_). The negative Curie–Weiss temperatures (−90, −30, −122, and −174 K) indicate that AFM interactions outcompete the FM interactions between V^*n*+^ centers in this temperature range.

The temperature-dependent neutron diffraction patterns for λ-Li_1_V_2_O_5_ (Supporting Figure S17) reveal no new reflections at low temperatures, indicating that magnetic order is weak or short-range. By contrast, the neutron diffraction patterns of λ-K_0.66_V_2_O_5_ (Figure 6C) display new reflections below 190 K, somewhat higher than the transition temperature indicated by magnetometry. These new reflections are relatively weak in intensity, consistent with the dilute magnetic moments expected from the stoichiometry of the ions and the respective electron localization on the [V_4_O_10_] frameworks. The V^4+^ magnetic moments being present on a minority of the vanadium sites is consistent with our proposed model of partial reduction, as seen in the HAXPES spectra provided in Figure 3. An antiferromagnetic structure model is inferred, characterized by the propagation vector ***k*** = 0 and the irreducible representation Γ_2_, which features *S* = 1/2 moments on half the vanadium sites, roughly consistent with the donation of 1/3 electron per vanadium from K^+^ cations into the V_2_O_5_ layers; this will be the focus of further work.

To understand the origin of the competing FM–AFM interactions, we have further investigated the structural and magnetic properties of λ-LiV_2_O_5_, λ-K_0.66_V_2_O_5_, and λ-Rb_0.5_V_2_O_5_ using DFT+U calculations as described in the Experimental Procedures. For each composition, we constructed supercells, illustrated in Supporting Figure S18 (altering the stoichiometry of λ-K_0.66_V_2_O_5_ to K_0.75_V_2_O_5_) in ferromagnetic (FM) and antiferromagnetic (AFM) configurations to determine the corresponding magnetic ground state. Our DFT+U calculations indicate that λ-LiV_2_O_5_ and λ-K_0.75_V_2_O_5_ favor an FM ground state, whereas λ-Rb_0.5_V_2_O_5_ tends to stabilize in an AFM configuration. However, the energy differences between the FM and AFM competing magnetic states are exceedingly small for all three compounds. More specifically, for their full supercell containing 16 V atoms, the total energy difference between the FM and AFM states is on the order of only tens of meV, corresponding to an average energy difference of about a few meV/atom or less.

The magnetic moment distributions also exhibit distinct characteristics among λ-LiV_2_O_5_, λ-K_0.75_V_2_O_5_, and λ-Rb_0.5_V_2_O_5_, as shown in Supporting Figure S19. Each supercell contains two sublayers consisting of 16 total atoms with eight V atoms in the upper layer and the other eight V atoms in the lower layer. In λ-LiV_2_O_5_, two types of V sites emerge, exhibiting markedly different magnetic moments (1 μ_B_ versus ∼0.1 μ_B_). The red arrows correspond to nearly nonmagnetic sites (0.12–0.14 μ_B_), whereas the black arrows denote V atoms with magnetic moments of around 1 μ_B_. In the case of λ-K_0.75_V_2_O_5_, three types of V atoms appear: two analogous to the Li case, and a third type with even smaller magnetic moments (0.03–0.09 μ_B_) as indicated by blue arrows. λ-Rb_0.5_V_2_O_5_ shows the strongest variation with four types of V sites: one carries a relatively large moment, whereas the remaining three exhibit rather small magnetic moments, as reflected in the arrow lengths.

The calculations above highlight the intrinsic complexity of the λ-M_*x*_V_2_O_5_ magnetic structure. λ-LiV_2_O_5_ and λ-K_0.75_V_2_O_5_ show a slightly greater propensity for FM interactions, whereas λ-Rb_0.5_V_2_O_5_ has a slight preference for an AFM state. However, the energy differences between these magnetic configurations are exceedingly small, which is concordant with experimental observations that indicate a close competition between the FM and AFM states.

We consider two possible structural origins of the variation in the strength of magnetic interactions. First, either static disorder (i.e., occupancy disorder between adjacent interstitial sites) or dynamic disorder (underpinned by rapid ion migration between adjacent sites such as split-site disorder and concomitant cation shuttling or superlattice ordering) may disrupt the patterns of electron localization on reduced vanadium sites. Indeed, in ε-Cu_0.9_V_2_O_5_, the magnetic transition occurs at or immediately below the transition temperature corresponding to the melting of superlattice ordering of Cu ions.^44^

To further probe ion reordering, we conducted inelastic neutron scattering measurements on ε-Cu_0.9_V_2_O_5_. The extracted phonon density of states is visible in Supporting Figure S20. Figure 6D,E depicts a low-energy phonon mode that almost entirely vanishes above the structural/magnetic transition, which implies that structural disorder associated with Cu-ion motion disrupts the coherence of long-range interactions. The lack of long-range magnetic ordering in λ-LiV_2_O_5_ may also be a result of the high mobility of the guest ion. Indeed, in single-crystal structure solutions of both Cu_0.9_V_2_O_5_ and λ-LiV_2_O_5_,^44,47^ large oblate thermal ellipsoids are observed for the intercalated ions, suggesting considerable Cu- and Li-ion thermal motion. The absence of a magnetic transition in λ-Na_0.83_V_2_O_5_, which shares the structure of ε-Cu_0.9_V_2_O_5_, is possibly a result of the significant disorder of intercalated sodium ions, evidenced by the weakness of modulation satellites observed in Supporting Figure S11 and discussed above.

Additional insight into guest ion mobility can be gleaned from the relative rates of insertion and removal reactions. Oxidative removal of Cu ions by NO_2_BF_4_ produces NO_2_ gas, the evolution of which serves as a visible indicator of reaction progress. Under the reaction conditions noted below, visible gas evolution ceases 5 s after the addition of ε-Cu_0.9_V_2_O_5_ to the dispersion, corresponding to the migration of the majority of Cu ions out of the bulk of ca. 10 μm particles. Ion insertion reactions also yield similar insight. Reductive insertion of M ions via their iodide salts generates I_2_ and I_3_^–^, resulting in a dark brown coloration indicative of reaction progress. The rate of the reaction, assessed by the rate of supernatant solution darkening, depends strongly on the guest ion identity. Li inserts within 10 s, whereas Rb and Cs require approximately 3 h to insert within λ-V_2_O_5_ to yield dispersions of comparable opacity.

To assess the ability of particular guest ions to induce or disrupt electron localization on nearby vanadium sites, we calculated electron localization function (ELF) maps for the λ-M_*x*_V_2_O_5_ materials (Figure 7A–E), which show significant distortion of the vanadyl oxygen electron density toward guest ions. The ELF maps in Figure 7A–E corroborate that the majority of the localized electron density near the Fermi level resides on oxygen ions. Although the electron density localized on vanadyl oxygens is visibly polarized toward the positively charged interlayer space in all materials, the direction and magnitude of this distortion vary significantly. Particularly for smaller and harder ions like Li^+^ and Na^+^, the electron density is distorted toward individual guest ions, whereas for larger and relatively more polarizable species such as Rb^+^ and Cs^+^, the electron density is polarized more indirectly toward the interstitial space, rendering the electronic structure within [V_4_O_10_] slabs more sensitive to the motion of small guest ions. As such, the specific arrangement of guest ions and [V_4_O_10_]–guest cation coupling mediated by vanadyl (V–O–M) interactions profoundly affect sliding and interlayer shear relationships.

**Figure 7:**
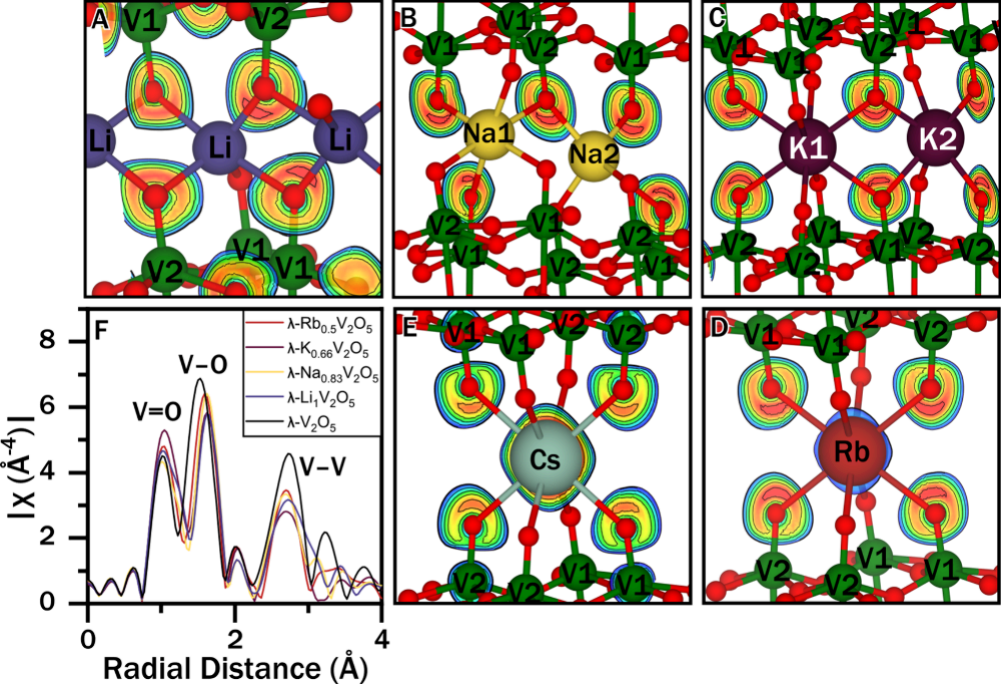
ELF maps and EXAFS fitting results. (A–E) ELF map slices for (A) λ-Li_1_V_2_O_5_, (B) λ-Na_0.83_V_2_O_5_, (C) λ-K_0.66_V_2_O_5_, (D) λ-Rb_0.5_V_2_O_5_, and (E) λ-Cs_0.5_V_2_O_5_, showing polarization of the vanadyl O^2–^ electron density along M–O bonds for smaller ions. (F) χ­(*R*) plot calculated from EXAFS spectra. Smaller ions lead to longer V–O bonds and a larger variation in V–V bond lengths.

The second structural contribution to magnetic transition temperature variation occurs via distortions induced by guest ions to the V_2_O_5_ host structure, particularly as they modify J-coupling along the V1–O–V2 superexchange pathways. Because Rietveld refinement of diffraction data provides an average structure and is somewhat limited in probing occupancy disorder, we fit the EXAFS spectra in Figure 3A to structural models to obtain local structure information. Figure 7F shows the magnitude of the Fourier transform of the *k* ^3^-weighted V K-edge EXAFS spectra of λ-V_2_O_5_ and λ-M_*x*_V_2_O_5_ (M = Li, Na, K, Rb). Additional fitting details are presented in the Supporting Information, Figure S21 and Tables S11–S15. The first coordination shell of vanadium manifests features centered at 1.50–1.75 Å. The narrowing and shift of these features to longer correlations with increasing intercalant concentration confirm the higher symmetry and increase in V–O bond lengths, respectively, with increasing intercalant concentration, which reflects the increasing reduction of vanadyl moieties as also inferred from the X-ray absorption spectroscopy measurements in Figure 3. Likewise, the broadening of the feature at 2.75 Å with increasing intercalant concentration as well as decreasing ionic radius indicates a broader distribution of V1–V2 correlations within the [V_4_O_10_] slabs. This trend is evident in the V1–V2 distances presented in Supporting Tables S11–S15.

Similar magnetic transition behavior has been observed in single crystals of ε-Cu_0.9_V_2_O_5_,^44^ δ-Na_0.5_V_2_O_5_, δ-Sr_0.5_V_2_O_5_,^40^ and δ-Tl_0.5_V_2_O_5_,^39^ accompanied by resistivity anomalies in each case. In ε-Cu_0.9_V_2_O_5_ and δ-K_0.5_V_2_O_5_, resistivity transitions are of sufficient magnitude to instantiate negative differential resistance,^42–44^ a sufficient condition for the materials to serve as active elements in neuron-emulative devices.^83^ We posit that these behaviors are specific manifestations of a close coupling, shared among the double-layered bronzes, between guest ion order, ion–lattice interactions, and charge localization and ordering.^37,84^ While this article focuses on deciphering the structural chemistry, identifying versatile synthetic routes for topochemical modification, and mapping shear relationships to electronic and magnetic structure, it is notable that ion insertion in these compounds positions the systems at the cusp of V–V distances where insulator–metal transitions can be induced as per Goodenough’s analysis.^85^ As such, the structural chemistry and ion modulation of V–V interactions, charge delocalization, and exchange interaction pathways have strong implications for accessing a rich class of metal–insulator transition materials that are much sought after for neuromorphic computing.

## Conclusion

In this work, we highlight topochemical ion insertion as a means of interlocking layered materials with well-defined and discretized shear relationships governed by coordination site preferences of Group I cations. We synthesized five such materials with M = {Li, Na, K, Rb, Cs} and determined their atomic structure from powder X-ray diffraction and scanning transmission electron microscopy. Intercalated ions construct their coordination sites by shearing two-dimensional V_2_O_5_ layers to align vanadyl oxo anions on slab surfaces. We develop a taxonomy of double-layered bronzes, which are classified as per seven observed guest ion coordination geometries with coordination numbers ranging from four to eight, which, in turn, yields four accessible discretized shear regimes separated in increments of half octahedral widths. X-ray absorption and emission spectroscopies reveal that frontier V 3d_*xy*_ band filling is proportional to intercalated ion stoichiometry, which suggests the site-selective topochemical or electrochemical positioning of cations as a means of controlling electronic structure. Comparison of magnetic susceptibility and neutron diffraction measurements, DFT and ELF calculations, and structure trends observed via PXRD and EXAFS fitting suggests that increasing guest ion radius weakens electrostatic guest–host interactions and decreases V1–V2 distances, strengthening magnetic coupling and stabilizing local magnetic interactions. Detailed analysis of the magnetic structure, which is challenging given the low symmetries and weak moments, will be the subject of future work. Such measurements will benefit from the growth of high-quality single crystals as is the focus of ongoing work.^37^ Overall, our results suggest that the selection of the intercalated cation and its stoichiometry provides a rich toolset for stitching together vertical stacks of 3D materials with well-defined interlayer shear relationships. The lower symmetry of the materials under consideration allows for clean isolation of distinctive and discretized shear relationships and their functional implications; the connection of shear transformations to ion composition and stoichiometry represents a key elaboration of concepts of chemical pressure. The clear effect that guest ions have on electron–electron interactions suggests the modulation of guest ion selection, stoichiometry, and positioning as a means of controlling electronic phase transition characteristics in strongly correlated materials.

## Experimental Procedures

### Synthesis of ε-Cu_0.9_V_2_O_5_

Stoichiometric amounts of copper metal powder (Aldrich, 99.5%) and V_2_O_5_ powder (Aldrich, ≥99.6%) were ground together in a mortar and pestle, sealed in a fused silica ampule under vacuum, and heated for 18 h at 550 °C in a Thermo Fisher Thermalyne F47900 muffle furnace. After cooling to ambient temperature, the ampules were opened and the black powder contents ground, sealed, and heated as previously described for another 18 h. The resulting black powder product was stored in air.

### Synthesis of λ-V_2_O_5_

ε-Cu_0.9_V_2_O_5_ precursor powder and all glassware were held under vacuum at 120 °C overnight to remove moisture. In an Ar-filled glovebox, the ε-Cu_0.9_V_2_O_5_ powder was added to a saturated solution of nitronium tetrafluoroborate (Fisher 97%) in ultradry acetonitrile (Thermo Fisher 99.9% Extra Dry over Molecular Sieves) at a 4:1 (mol/mol) NO_2_BF_4_:ε-Cu_0.9_V_2_O_5_ ratio. In an example reaction, 1.0079 g of ε-Cu_0.9_V_2_O_5_ and 2.2031 g of NO_2_BF_4_ were reacted in 25 mL of acetonitrile. The mixture reacted vigorously, producing brown gas and yielding a blue supernatant color suggesting the leaching of Cu ions. After off-gassing subsided, the dispersion was stirred gently for 3 h and decanted to recover the yellow powder product. The product was washed with copious amounts of acetonitrile and dried overnight under argon at 70 °C on a hot plate. The resulting yellow λ-V_2_O_5_ powder product was stored under argon.

### Topochemical Ion Intercalation

λ-V_2_O_5_ powder and a ca. 2:1 stoichiometric molar excess of alkali-metal iodide salt (LiI: Sigma-Aldrich 99.9%; NaI: Sigma-Aldrich ≥99.5%; KI: Sigma-Aldrich ReagentPlus 99%; RbI: Sigma-Aldrich 99.9%; CsI: Sigma-Aldrich 99.9%) were stirred together in acetonitrile for 24 h under argon. As an example, 402.7 mg of dried λ-V_2_O_5_ was mixed with 619.1 mg of NaI in approximately 3.5 mL of acetonitrile. The apparent reaction rate (as indicated by darkening of the supernatant and powder) followed the trend of alkali-metal atomic number, with the LiI solution reacting immediately and the CsI solution reacting only after several hours, likely also reflecting differences in the solubility of metal iodides. The products were allowed to settle, decanted, washed, and dried as described above. The black powder products were stored under argon.

### Laboratory Powder X-ray Diffraction

Laboratory powder X-ray diffraction was performed on a Bruker D8 Eco diffractometer in the Bragg–Brentano configuration, equipped with a Cu K_α_ anode X-ray source (λ = 1.5418 Å) and a Lynxeye detector, with a 40 kV voltage and 25 mA current. Air-sensitive samples were mounted in sample holders in a glovebox under argon and sealed for measurement using a screw-on plastic dome. Rietveld refinements of diffraction data were performed using GSAS-II structure analysis software.^86^

### Scanning Electron Microscopy and Energy-Dispersive X-ray Spectroscopy

Scanning electron microscopy images and energy-dispersive X-ray (EDX) spectra were collected at a 20 kV accelerating voltage using a JEOL JSM-7500F FE (RRID: SCR_022202) instrument equipped with an Oxford Instruments Ultim Max40 silicon drift detector from samples gently pressed onto carbon tape.

### Scanning Transmission Electron Microscopy

Scanning transmission electron microscopy (STEM) imaging was performed on a probe-corrected TFS Spectra 200 instrument operating at 200 kV with a 24.2 mrad convergence semiangle. A low beam current of 15 pA was used to minimize the beam damage. High-angle annular dark-field (HAADF)-STEM images were acquired using inner and outer collection angles of 56 and 200 mrad, respectively. TEM lamellae were prepared from powder particles using a Tescan Solaris Ga-FIB following standard focused ion beam (FIB) liftout procedures.

### Synchrotron Powder X-ray Diffraction

Synchrotron powder X-ray diffraction measurements on λ-M_*x*_V_2_O_5_ (M = Li, Na, K, Rb; Supporting Figures S3–S6) were conducted at the QAS beamline (7-BM) at the National Synchrotron Light Source II at Brookhaven National Laboratory. Diffraction patterns were recorded with a large-area amorphous silicon detector (PerkinElmer 1621), featuring 2048 × 2048 pixels with a 200 × 200 μm pixel size. A LaB_6_ standard was utilized for detector calibration. Rietveld refinement was performed using GSAS-II software.^86^

Synchrotron powder X-ray diffraction data for λ_2_-V_2_O_5_ (Supporting Figure S2) were collected at beamline 11-BM at the Advanced Photon Source (APS) of Argonne National Laboratory during the 2023-1 run cycle. The specimen containing λ_2_-V_2_O_5_ powder mixed with carbon black, graphite, and PTFE binder in a weight ratio of 70:7.5:7.5:15 was pressed into a 9 mm diameter pellet and assembled in a custom AMPIX cell^87^ with a glass fiber separator and lithium metal anode. The electrolyte was a 1 M LiPF_6_ solution in a 1:1 (v/v) mixture of ethylene carbonate and diethylene carbonate. X-ray diffraction was performed in transmission mode with an X-ray wavelength of 0.4597 Å. Instrumental parameters were calibrated using LaB_6_ standards. Individual diffraction patterns were collected by using a 12-analyzer detector system that combines independent Si(111) crystal analyzers with LaCl_3_ scintillation detectors, and the data were subsequently merged and processed. The integrated λ-V_2_O_5_ powder pattern was Rietveld-refined using GSAS-II.

### Hard X-ray Photoelectron Spectroscopy

HAXPES measurements were performed at National Institutes of Standards and Technology (NIST) beamline 7-ID-2 at the National Synchrotron Light Source II (NSLS-II) at Brookhaven National Laboratory. X-ray energies were selected by using a Si(111) double-crystal monochromator. Spectra were collected at a 2 keV excitation energy using a hemispherical electron energy analyzer oriented perpendicular to the beam axis with a 500 eV pass energy and 50 meV step size. The beam spot position on samples and electron flood gun current and voltage were carefully controlled to mitigate sample charging.

### X-ray Absorption Near-Edge Structure Spectroscopy

XANES measurements were completed at NIST beamline 7-ID-1 at NSLS-II. The incident beam energy was selected using a variable line spacing plane grating monochromator. An electron flood gun was used to prevent sample charging. Partial electron yield signals were collected using a channeltron electron multiplier under a −300 V detector entrance grid bias and normalized to the incident beam intensity, as measured by a freshly evaporated gold mesh. Spectra were pre-to-post-edge normalized and energy-aligned to the V 3d_*xy*_ pre-edge feature of a fresh α-V_2_O_5_ standard (Aldrich, ≥99.6%) using the ATHENA software package.^88^

### Extended X-ray Absorption Fine Structure Spectroscopy

V K-edge X-ray absorption spectroscopy (XAS) scans were acquired at beamline 7-BM at the National Synchrotron Light Source II of Brookhaven National Laboratory. Samples were prepared by uniformly spreading the powder onto a piece of polyimide (Kapton) tape. The polyimide tape was then loaded onto a sample holder, and 20 scans were performed at 30 s per scan and subsequently averaged to improve the signal-to-noise ratio. Before sample acquisition, the beamline was calibrated by placing metallic vanadium, copper, and lead foils and measuring the edge position. Spectra were collected in both fluorescent and transmittance modes. The ATHENA program from the IFEFFIT package was used for data sanitization.^88^ Data in the *k* range of 3 to 13 Å^–1^ were Fourier transformed to obtain *R*-space data. The *R*-space data were used to perform shell fitting. Fitting was performed for the major shells between *R*-space = 1.1 and 4.0 Å. Multishell least-squares parameter fitting of V K-edge and Cu K-edge EXAFS data was performed using the ARTEMIS module of the IFEFFIT software package.^88^ The photoelectron mean free path, scattering amplitude, and phase functions were calculated by using the FEFF6 program. Atomic coordinates and lattice parameters obtained from crystallographic data were used to build initial models for EXAFS fitting. The Fourier transform was performed in the *k* range of 3–13 Å^–1^ using a Hanning window not corrected for phase shifts.

### Density Functional Theory and Electron Localization Function Calculations

First-principles calculations were carried out by using density functional theory (DFT)^89,90^ as implemented in the Vienna *Ab initio* Simulation Package (VASP).^91,92^ We used the projector-augmented wave (PAW) method^93,94^ with the exchange-correlation energy functional in the Perdew–Burke–Ernzerhof form^95^ and a plane-wave basis with an energy cutoff of 500 eV, a maximum residual energy of less than 1 × 10^–5^ eV, and a maximum residual force of less than 1 × 10^–4^ eV/Å. A Monkhorst–Pack *k*-point sampling^96^ with a grid of 3 × 12 × 4 was applied. To properly take into account the weak van der Waals interaction, we adopted Grimme’s DFT-D3 method with zero-damping function.^97^

### Magnetic Susceptibility

Magnetic measurements were carried out on powders by using a Quantum Design Magnetic Property Measurement System using the Quantum Design superconducting quantum interference device (SQUID) magnetometer option. Both zero-field-cooled (ZFC) and field-cooled (FC) measurements were performed in the temperature range of 2 to 400 K with an applied field up to 0.1 T. Field-dependent magnetization measurements were performed at 2 K and above room temperature under an applied magnetic field ranging from −7 to +7 T.

### Exfoliation and SEM

Single crystals of λ-V_2_O_5_ and λ-K_0.63_V_2_O_5_ were made from ε-Cu_0.9_V_2_O_5_ single crystals using a method similar to that employed in the literature.^47^ The as prepared single crystals were affixed to an SEM stub using carbon tape with the largest face (corresponding to the 001 plane) aligned parallel to the tape surface. Crystals were mechanically exfoliated by pressing a second piece of carbon tape onto the top of the crystals using a spatula and pulling the tape off. This procedure was repeated four times with small lateral movements between applications to spread the exfoliated sheets across the carbon tape. The exfoliated crystals were imaged in a field emission gun equipped scanning electron microscope (SEM) Zeiss ULTRA 55 using an In-lens detector with a 3.6 kV accelerating voltage and 6.3 mm working distance.

### Variable Temperature Powder X-ray Diffraction

λ-Cs_0.5_V_2_O_5_ was allowed to hydrate in air for 7 days. Temperature-dependent powder X-ray diffraction was conducted on λ-Cs_0.5_V_2_O_5_ using an Anton Parr high temperature 1200N oven and PANalytical Empyrean diffractometer with Cu K_α_ radiation (λ = 1.5406 Å) equipped with an Anton Parr high temperature 1200N oven with a ZrO_2_/Al_2_O_3_ sample stage. Samples were annealed for 1 h and then measured for 2 h at each temperature from 25 to 300 °C, as shown in Supporting Figure S9.

### Magnetism Sample Preparation for λ-Cs_0.5_V_2_O_5_

A 40 mg aliquot of λ-Cs_0.5_V_2_O_5_ powder was transferred into a quartz ampule, evacuated, backfilled with high-purity argon in a glovebox, and flame-sealed to avoid any exposure to moisture. The as packed sample was measured on a Quantum Design Magnetic Property Measurement System using the Quantum Design superconducting quantum interference device (SQUID) magnetometer option. Both zero-field-cooled (ZFC) and field-cooled (FC) measurements were performed in the temperature range of 2 to 375 K with an applied field up to 0.1 T. Field-dependent magnetization measurements were performed at 2 K and above room temperature under an applied magnetic field ranging from −7 to +7 T.

### Neutron Diffraction

Neutron powder diffraction measurements were performed on a high-intensity two-axis cold neutron diffractometer, DMC, at the Swiss Spallation Neutron Source, SINQ, with a calibrated incident wavelength of 2.455 Å. Samples were packed in a helium glovebox into cylindrical thin-walled vanadium sample cans (λ-K_0.66_V_2_O_5_: ∼8 mm diameter; λ-LiV_2_O_5_: ∼6 mm diameter) and sealed with indium O-rings.

### Inelastic Neutron Scattering

Powder inelastic neutron scattering was performed using the time-of-flight direct geometry spectrometer FOCUS at PSI. An incident neutron wavelength of 4 Å was used for all measurements. Spectra were acquired for a temperature range from 140 to 300 K. Measurements with long counting times were carried out at 140, 160, and 300 K, while short acquisition times were measured while warming from 140 to 160 K. Data reduction was performed with DAVE.^98^

### M_*x*_V_2_O_5_ Shear Calculation Methods

Energy versus Δ curves were constructed via DFT using the same parameters as described in the Density Functional Theory and Electron Localization Function Calculations section. Theoretical concentrations of guest ions were created by selectively filling guest ion sites with respect to fractional occupancy found via experiment and then fully relaxing the structure. Individual sheared structures along each curve were constructed from each relaxed structure by manually changing the *c* lattice parameter and monoclinic angle β while holding the interlayer spacing and spatial positions of atoms fixed to generate shear along the in-plane direction *a* in increments of 0.1 Å. Structures were then optimized by allowing all atoms to relax but keeping the lattice shape and volume fixed. Results were plotted with respect to the lowest-energy structure (corresponding to the ground state). Plots were shifted horizontally to situate the energy minima at 0 shear.

### Magnetic Configuration Calculations

For magnetic state calculations, we first constructed supercells for λ-LiV_2_O_5_, λ-K_0.75_V_2_O_5_, and λ-Rb_0.5_V_2_O_5_ in order to accommodate the various possible magnetic configurations. Next, we constructed various magnetic configurations for each compound in the supercell described above, including the FM state and a set of AFM configurations with distinct spin patterns on the V atom sublattice. Each configuration was subsequently optimized by allowing the atomic coordinates to fully relax while keeping the supercell lattice vectors fixed. We then carried out electronic self-consistent field calculations for each optimized configuration and obtained the total energies as well as the magnetic moments on each V atom. By examining both the total energy differences and the resulting magnetic moments among the FM and various AFM states, we identified the most energetically favorable magnetic ground state for each compound.

### Exfoliation Energy Calculations

Exfoliation energy is calculated via VASP calculations using the formalism described by Jung et al.^99^

$$E_{\mathrm{exfoliation}}=\frac{E_{\mathrm{isolated layer}}-E_{\mathrm{bulk}}}{A}$$

which takes the exfoliation energy *E*_exfoliation_ as the difference between the ground state energy per layer of the bulk material, *E*_bulk_, and the energy of one isolated layer in a vacuum, *E*_isolated layer_. The in-plane cross-sectional area of the bulk unit cell is denoted by *A*. The lattice shape and volume of the isolated layer were held constant during relaxation.

## Supplementary Material



## Notes

Additional experimental results, including X-ray and neutron diffraction patterns and refinement tables, SEM images and EDX composition maps, structure comparison tables, schematic diagrams, additional magnetometry measurements, calculation results, inelastic neutron scattering data, and EXAFS fitting results (PDF)
