# Supplementary material for: Alkali-Metal Interlocking of 2D V4O10 Sheets Defines Discretized Interlayer Shear Relationships
Source: J Am Chem Soc. 2026 Feb 19;148(8):8174–89. doi: 10.1021/jacs.5c16903 (PMC12964412; doi:10.1021/jacs.5c16903)
Supplement: Supplementary file 1 [file ja5c16903_si_001.pdf]

Supporting Information

Alkali-Metal Interlocking of 2D V_4O_{10} Sheets Defines Discretized Interlayer Shear Relationships

*John Ponis,[1§] Kenna Ashen,[2§] Sarbajeet Chakraborty,[3,4§] George Agbeworvi,[1,2] Michelle A. Smeaton,[5] Chengdong Wang,[2] Amanda Jessel,[3,4] Douglas H. Fabini,[6] Fanni Juranyi,[7] Diana Quintero-Castro,[7] Nick A. Shepelin,[6] Dariusz Jakub Gawryluk,[6] Katherine L. Jungjohann,[5] Shruti Hariyani,[1,2,*3] Xiaofeng Qian,[*2] Sarbajit Banerjee[2,3,*5]*

1. Department of Chemistry
Texas A&M University
College Station, Texas 77843, United States

2. Department of Materials Science and Engineering
Texas A&M University
College Station, Texas 77843, United States
E-mail: feng@tamu.edu

3. Laboratory for Battery Science, PSI Center for Energy and Environmental Sciences
Paul Scherrer Institute
Forschungsstrasse 111, CH-5232 Villigen PSI, Switzerland
E-mail: shruti.hariyani@psi.ch

4. Laboratory for Inorganic Chemistry, Department of Chemistry and Applied Biosciences
ETH Zurich
Vladimir-Prelog-Weg 2, CH-8093 Zürich, Switzerland
E-mail: sbanerje@ethz.ch

5. National Laboratory of the Rockies
Golden, Colorado 80401, United States

6. PSI Center for Neutron and Muon Sciences
Paul Scherrer Institute,
Forschungsstrasse 111, CH-5232 Villigen PSI, Switzerland

7. Laboratory for Neutron Scattering and Imaging, PSI Center for Neutron and Muon Sciences, Paul Scherrer Institute
Forschungsstrasse 111, 5232 Villigen PSI, Switzerland

§ *These authors have contributed equally to this work*

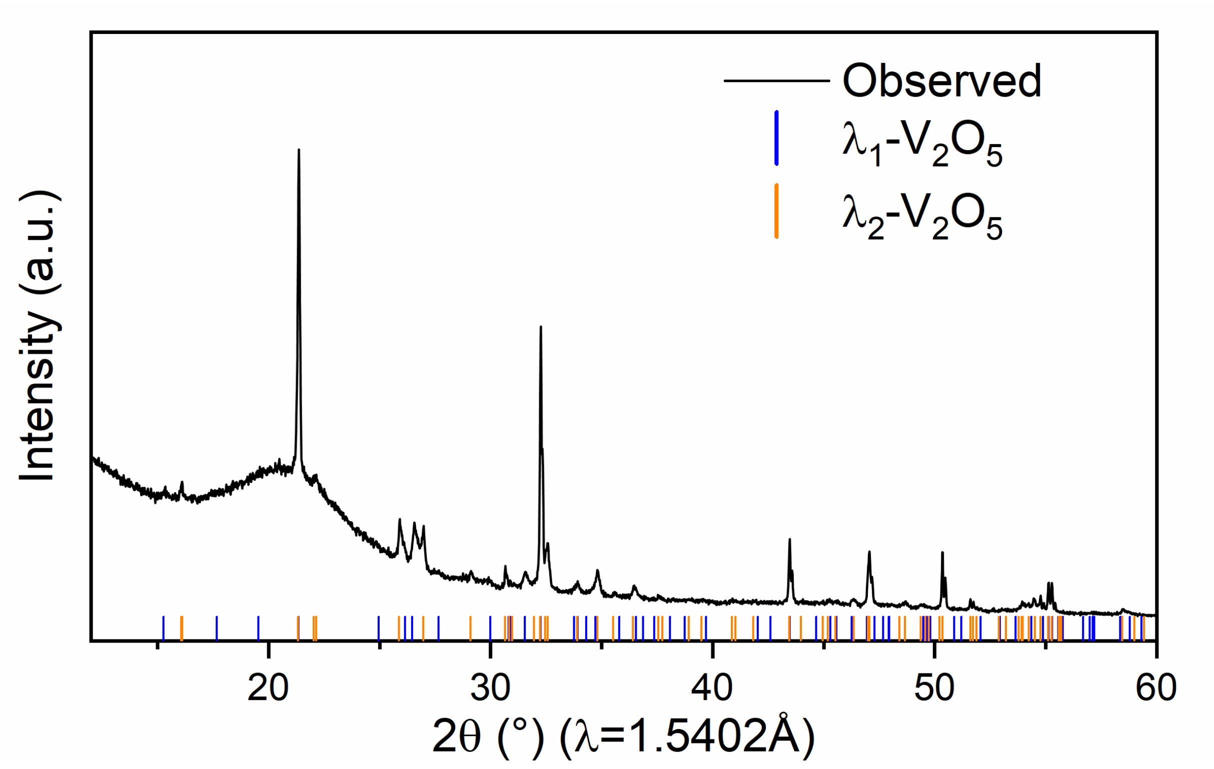

Figure S1: Powder X-ray diffraction pattern of as-synthesized λ-V_2O_5. Data collected on a Bruker D8 Advance diffractometer, with the large background signal originating from the air-sensitive sample holder. Reflections are indexed to λ_1-V_2O_5 and λ_2-V_2O_5, as indicated by vertical ticks.

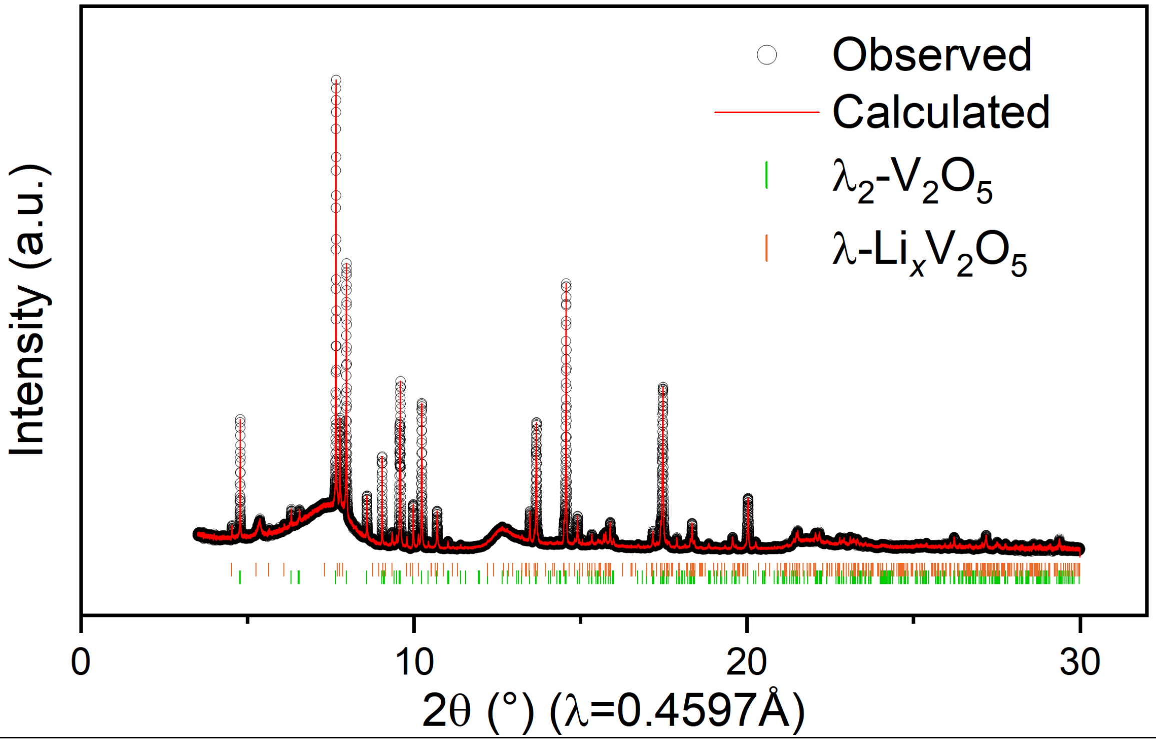

Figure S2: Rietveld refinement of λ_2-V_2O_5. Diffraction data collected at beamline 11-BM of the Advanced Light Source. Refinement statistics and structure results given in Tables S1 and S2.

Table S1: Refined lattice parameters, atomic positions and thermal parameters for λ_2-V_2O_5. Corresponding diffraction data shown in Figure S2.

λ_2-V_2O_5

**wR**	7.32%	**R**	5.77%	**χ^2**	2.1
**2ϑ range**	3.5-25.9978°	**Radiation**	Synchrotron, 0.4597Å	**Temp**	295 K
**Formula**	V_2O_5	**Z**	4	**wt.%**	85.311(95)%
a **(Å)**	11.6676(6)	***b*** **(Å)**	3.626388(19)	***c*** **(Å)**	8.8524(5)
β **(°)**	109.4749(13)	***V*** **(Å³)**	353.128(9)	**S.G.**	*C*2/*m*

**Atom**	**x**	**y**	**z**	**frac**	**Wyckoff**	**Uiso**
**V1**	0.48210(14)	0.5	0.6822(4)	1	4*i*	0.0097(5)
**V2**	0.77361(13)	0.5	0.6863(4)	1	4*i*	0.0102(5)
**O1**	0.5399(5)	0.5	0.8738(12)	1	4*i*	0.0471(27)
**O2**	0.7881(5)	0.5	0.8650(12)	1	4*i*	0.0337(22)
**O3**	0.4302(4)	0	0.6574(9)	1	4*i*	0.0018(12)

**O4**	0.7862(4)	0	0.6469(11)	1	4*i*	0.0044(14)
**O5**	0.6108(5)	0.5	0.6122(12)	1	4*i*	0.0266(18)

Table S2: Refined lattice parameters, atomic positions and thermal parameters for λ-$Li_xV_2O_5$. λ-$Li_xV_2O_5$ likely formed during cell preparation for *operando* powder diffraction via Li insertion from the $LiPF_6$ electrolyte into $λ_1$-V_2O_5 and/or $λ_2$-V_2O_5. As the purpose of this refininement is to determine the structure of $λ_2$-V_2O_5, the similar structure of $λ_1$-V_2O_5 (CCDC 2154907) was used as a basis, only vanadium atomic positions were refined.

λ-$Li_xV_2O_5$					
**wR**	7.32%	**R**	5.77%	**$Χ^2$**	2.1
**2ϑ range**	3.5-25.9978°	**Radiation**	Synchrotron, 0.4597Å	**Temp**	295 K
**Formula**	V_2O_5	**Z**	4	**wt.%**	14.689(95)%
a **(Å)**	11.669(11)	***b*** **(Å)**	3.64016(8)	***c*** **(Å)**	10.078(10)
β **(°)**	120.898(18)	***V*** **(Å³)**	367.33(7)	**S.G.**	*C2/m*

**Atom**	**x**	**y**	**z**	**frac**	**Wyckoff**	**Uiso**
**V1**	0.81448	0.5	0.70616	1	4*i*	0.0088
**V2**	0.49399	0.5	0.63346	1	4*i*	0.0088
**O1**	0.8176	0	0.6359	1	4*i*	0.0141
**O2**	0.8668	0.5	0.8715	1	4*i*	0.0141
**O3**	0.6255	0.5	0.6082	1	4*i*	0.0141
**O4**	0.6072	0.5	0.8716	1	4*i*	0.0141
**O5**	0.4587	0	0.6506	1	4*i*	0.0141

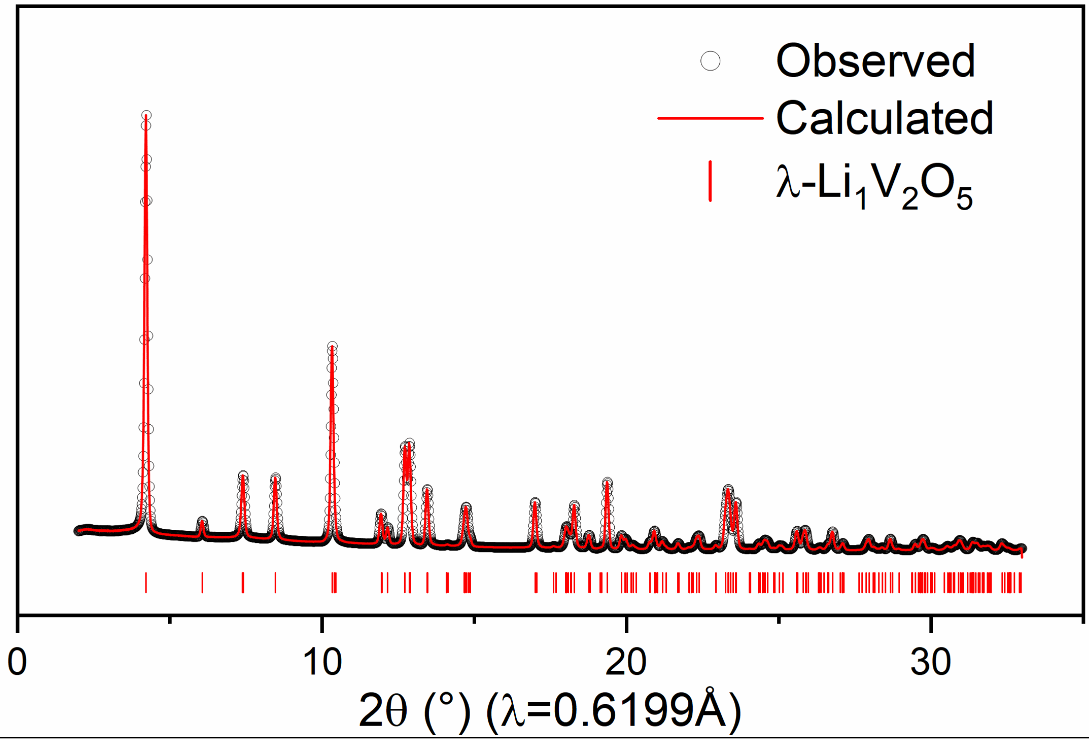

Figure S3: Powder X-ray diffraction pattern and Rietveld refinement for λ-$Li_1V_2O_5$. Diffraction data collected at beamline 7-BM of the National Synchrotron Light Source II. Refinement statistics and structure results are provided in Table S3.

Table S3: Refined lattice parameters, atomic positions and thermal parameters for λ-$Li_1V_2O_5$. Corresponding diffraction data shown in Figure S3.

λ-$Li_1V_2O_5$

**wR**	5.41%	**R**	3.97%	**X^2**	0.52
**2ϑ range**	2.5-32.9876°	**Radiation**	Synchrotron, 0.6199Å	**Temp**	295 K
**Formula**	$Li_1V_2O_5$	**Z**	4		
a **(Å)**	11.695(4)	***b*** **(Å)**	3.68161(10)	***c*** **(Å)**	10.2414(30)
β **(°)**	125.062(6)	***V*** **(Å³)**	360.930(24)	**S.G.**	*C*2/*m*

**Atom**	**x**	**y**	**z**	**frac**	**Wyckoff**	**Uiso**
**V1**	0.3466(3)	1	0.8271(4)	1	4*i*	0.0052(10)
**V2**	0.6468(3)	1	0.8363(4)	1	4*i*	0.0029(11)
**O1**	0.2572(10)	1	0.6314(11)	1	4*i*	0.026(4)
**O2**	0.5405(9)	1	0.6530(11)	1	4*i*	0.026(4)
**O3**	0.3279(12)	0.5	0.8539(8)	1	4*i*	0.024(3)

**O4**	0.7016(12)	0.5	0.8796(11)	1	4*i*	0.015(4)
**O5**	0.5231(11)	1	0.8950(10)	1	4*i*	0.020(4)
**Li1**	0.326(3)	1	0.463(4)	1	4*i*	0.010(8)

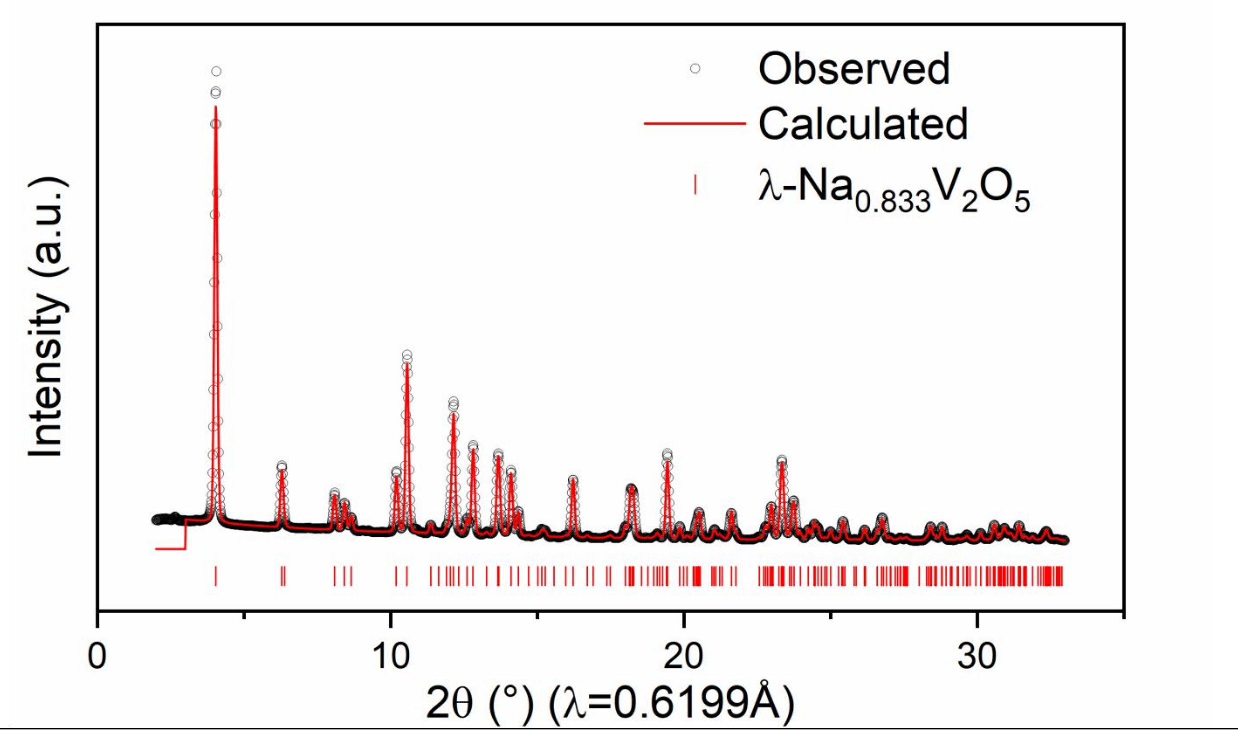

Figure S4: Powder X-ray diffraction pattern and Rietveld refinement of λ-$Na_{0.833}V_2O_5$. Diffraction data collected at beamline 7-BM of the National Synchrotron Light Source II. Refinement statistics and structure results given in Table S4.

Table S4: Refined lattice parameters, atomic positions and thermal parameters for λ-$Na_{0.833}V_2O_5$. Corresponding diffraction data shown in Figure S4.

**λ-$Na_{0.833}V_2O_5$**						
**wR**	4.72%	**R**	3.70%		**χ^2**	0.41
**2ϑ range**	3-32.9876˚	**Radiation**	Synchrotron, 0.6199Å		**Temp**	295 K
**Formula**	$Na_{0.833}V_2O_5$	**Z**	4			
a **(Å)**	11.8128(17)	***b*** **(Å)**	3.67124(8)		***c*** **(Å)**	9.3436(11)
β **(˚)**	109.929(3)	***V*** **(Å³)**	380.944(20)		**S.G.**	*C*2/*m*
**Atom**	**x**	**y**	**z**	**frac**	**Wyckoff**	**Uiso**
**V1**	0.52110(21)	1	0.83526(23)	1	4*i*	0.0061(9)

**V2**	0.22595(20)	1	0.84379(24)	1	4*i*	0.0054(9)
**O1**	0.4620(5)	1	0.6587(8)	1	4*i*	0.0211(24)
**O2**	0.1958(6)	1	0.6638(9)	1	4*i*	0.043(3)
**O3**	0.5603(6)	0.5	0.8650(8)	1	4*i*	0.0240(27)
**O4**	0.2021(6)	0.5	0.8873(6)	1	4*i*	0.0177(29)
**O5**	0.3953(6)	1	0.8936(8)	1	4*i*	0.0232(29)
**Na1**	0	1	0.5	0.883(13)	2*c*	0.041(4)
**Na2**	0.2773(12)	0.5	0.5502(14)	0.392(8)	4*i*	0.032(5)

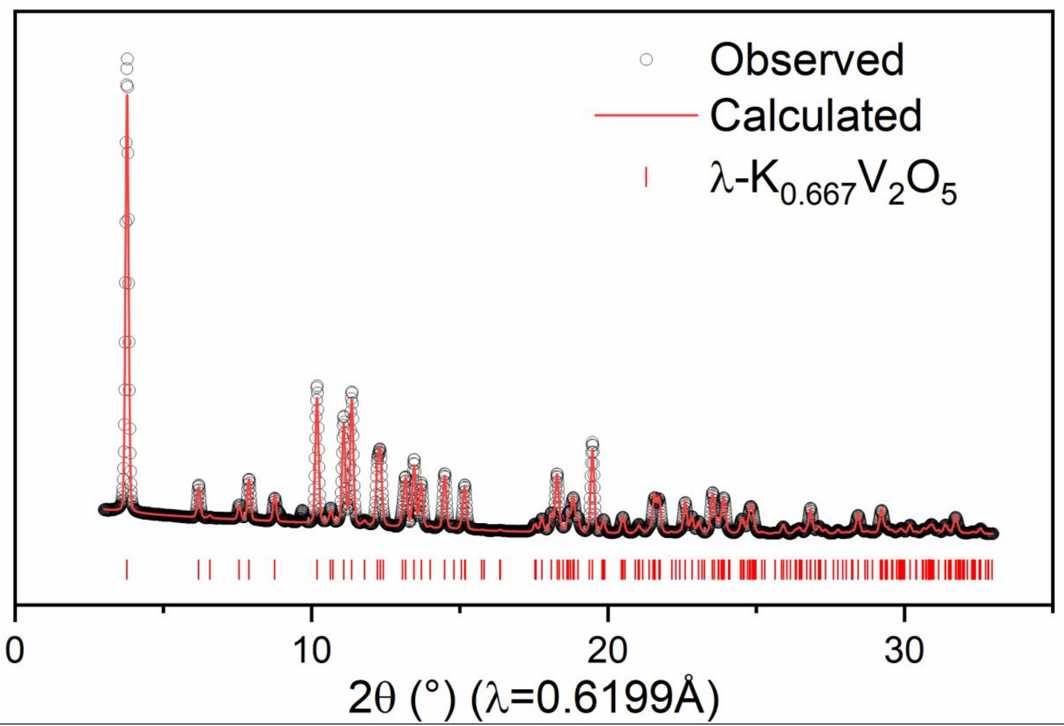

Figure S5: Powder X-ray diffraction pattern and Rietveld refinement of λ-$K_{0.667}V_2O_5$. Diffraction data collected at beamline 7-BM of the National Synchrotron Light Source II. Refinement statistics and structure results given in Table S5.

Table S5: Refined lattice parameters, atomic positions, and thermal parameters for λ-$K_{0.667}V_2O_5$. Corresponding diffraction data are shown in Figure S5.

λ-$K_{0.667}V_2O_5$					
**wR**	5.16%	**R**	3.56%	**X^2**	0.46
**2ϑ range**	3-32.9876°	**Radiation**	Synchrotron, 0.6199Å	**Temp**	295 K
**Formula**	$K_{0.667}V_2O_5$	**Z**	4		
a **(Å)**	11.7030(15)	***b*** **(Å)**	3.66350(10)	***c*** **(Å)**	9.5904(9)

β(°)	101.672(5)		V(Å³)	402.679(26)	S.G.	$C2/m$
**Atom**	**x**	**y**	**z**	**frac**	**Wyckoff**	**Uiso**
**V1**	0.08944(30)	0	0.15297(28)	1	4*i*	0.0028(10)
**V2**	0.29210(27)	0.5	0.15343(24)	1	4*i*	0.0085(12)
**O1**	0.1028(6)	0	0.3204(10)	1	4*i*	0.0199(29)
**O2**	0.3408(6)	0.5	0.3178(10)	1	4*i*	0.021(3)
**O3**	0.1137(12)	0.5	0.1347(9)	1	4*i*	0.0346(32)
**O4**	0.2468(10)	0	0.1102(8)	1	4*i*	0.0083(30)
**O5**	0.4326(10)	0.5	0.0948(8)	1	4*i*	0.0090(24)
**K1**	0	0.5	0.5	0.631(6)	2*c*	0.0184(28)
**K2**	0.1895(7)	0.5	0.5008(12)	0.351(4)	4*i*	0.017(4)

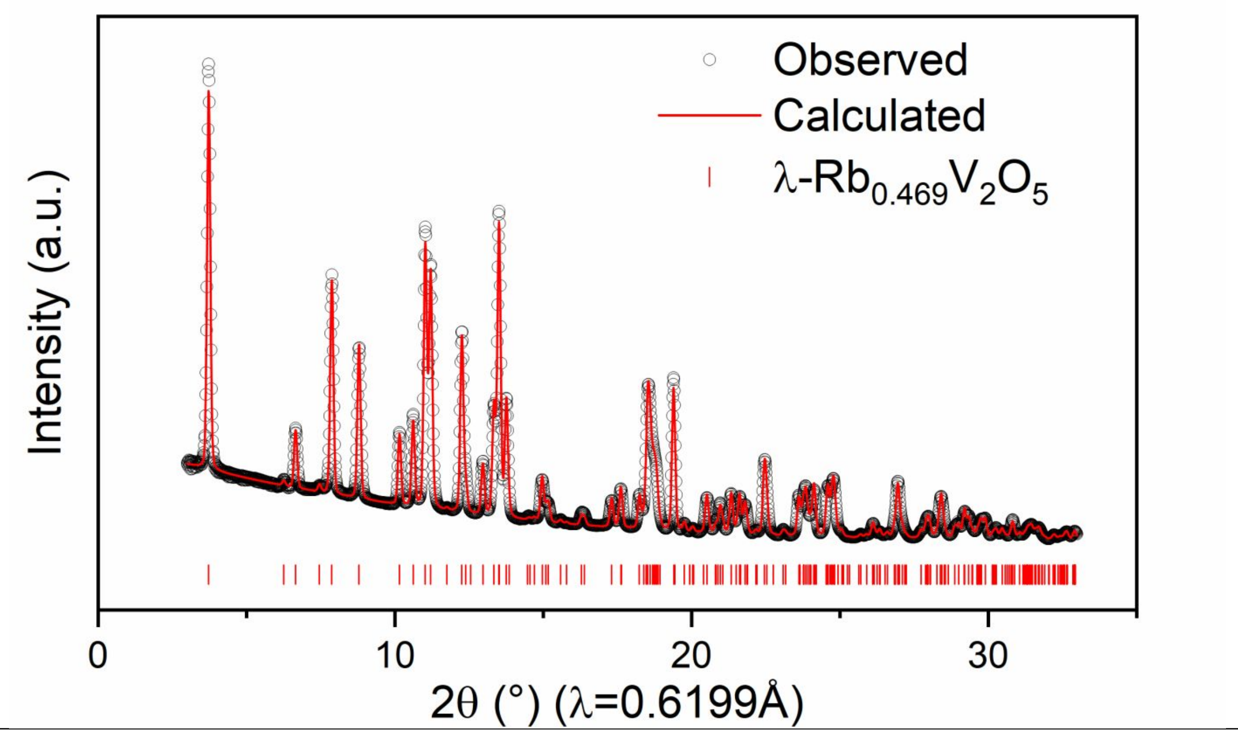

Figure S6: Powder X-ray diffraction pattern and Rietveld refinement of λ-$Rb_{0.469}V_2O_5$. Diffraction data collected at beamline 7-BM of the National Synchrotron Light Source II. Refinement statistics and structure results given in Table S6.

Table S6: Refined lattice parameters, atomic positions, and thermal parameters for λ-$Rb_{0.469}V_2O_5$. Corresponding diffraction data shown in Figure S6.

		λ-$Rb_{0.469}V_2O_5$			
**wR**	1.98%	**R**	1.52%	χ^2	0.11

**2ϑ range**	3-32.9876°	**Radiation**	Synchrotron, 0.6199Å	**Temp**	295 K
**Formula**	$Rb_{0.469}V_2O_5$	**Z**	4		
a **(Å)**	11.5469(6)	***b*** **(Å)**	3.67849(7)	***c*** **(Å)**	9.6913(5)
β **(°)**	100.9067(20)	***V*** **(Å^3)**	404.206(21)	**S.G.**	*C*2/*m*

Atom	x	y	z	frac	Wyckoff	Uiso
**V1**	0.40869(18)	0.5	0.84715(19)	1	4*i*	0.0061(7)
**V2**	0.20701(15)	0	0.84662(18)	1	4*i*	0.0068(7)
**O1**	0.3966(4)	0.5	0.6817(7)	1	4*i*	0.0249(21)
**O2**	0.1680(4)	0	0.6897(6)	1	4*i*	0.0152(20)
**O3**	0.3737(5)	0	0.8676(5)	1	4*i*	0.0077(18)
**O4**	0.2389(5)	0.5	0.8842(5)	1	4*i*	0.0215(19)
**O5**	0.0614(5)	0	0.9032(5)	1	4*i*	0.0024(15)
**Rb1**	0.5	0	0.5	0.9378(23)	2*c*	0.0141(7)

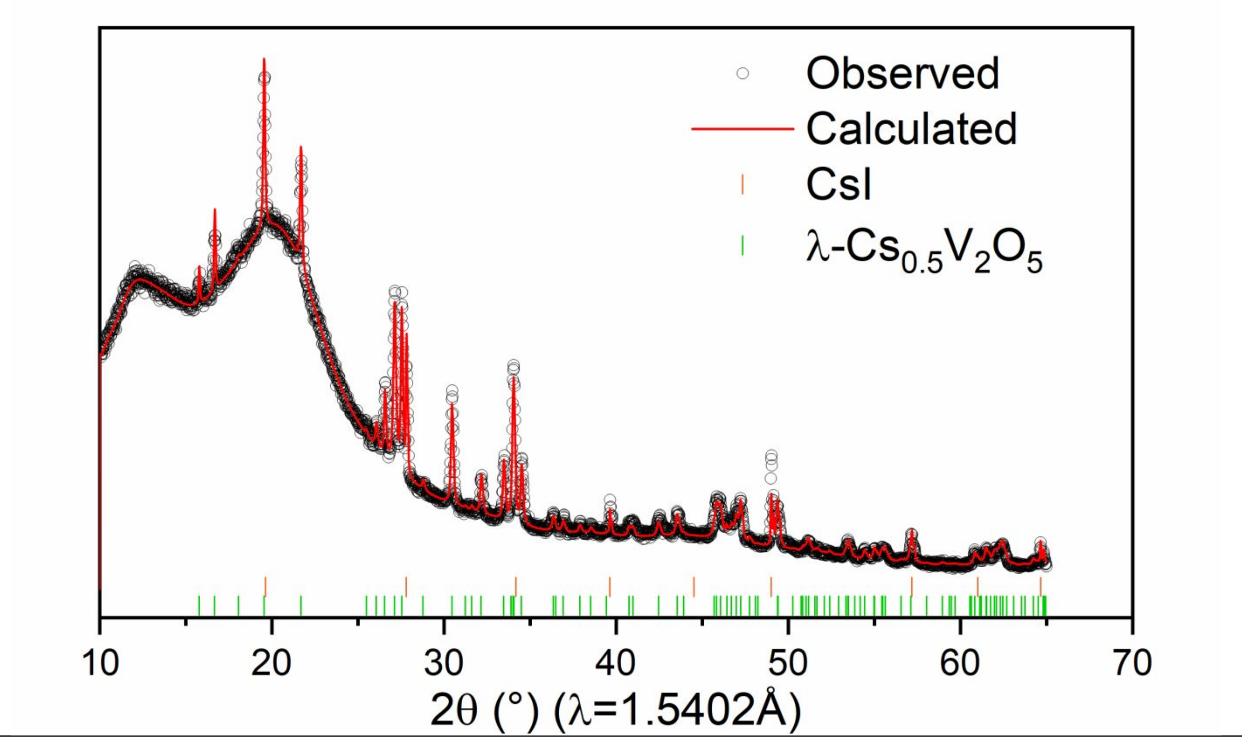

Figure S7: Powder X-ray diffraction pattern and Rietveld refinement of λ-$Cs_{0.5}V_2O_5$. Data collected on a Bruker D8 Advance diffractometer, with the large background signal originating from the air-sensitive sample holder. Refinement statistics and structure results given in Tables S7 and S8.

Table S7: Refined lattice parameters, atomic positions, and thermal parameters for λ-$Cs_{0.5}V_2O_5$. Corresponding diffraction data are shown in Figure S7.

λ-$Cs_{0.5}V_2O_5$

**wR**	3.41%	**R**	2.29%	**χ^2**	2.14
**2ϑ range**	10-64.9967°	**Radiation**	Cu Kα, 1.5402Å	**Temp**	295 K
**Formula**	$Cs_{0.5}V_2O_5$	**Z**	4	**wt.%**	97.038(66)
a **(Å)**	11.5831(14)	***b*** **(Å)**	3.70331(17)	***c*** **(Å)**	10.1050(11)
β **(°)**	100.548(5)	***V*** **(Å³)**	426.14(5)	**S.G.**	*C*2/*m*

**Atom**	**x**	**y**	**z**	**Frac**	**Wyckoff**	**Uiso**
**V1**	0.4080(9)	0.5	0.8540(7)	1	4*i*	0.0134(28)
**V2**	0.2081(9)	0	0.8533(9)	1	4*i*	0.034(4)
**O1**	0.3975(17)	0.5	0.6934(23)	1	4*i*	0.035(10)
**O2**	0.1774(22)	0	0.7075(31)	1	4*i*	0.059(12)

**O3**	0.3823(23)	0	0.8760(22)	1	4*i*	0.016(8)
**O4**	0.2321(23)	0.5	0.8923(23)	1	4*i*	0.056(10)
**O5**	0.0589(24)	0	0.9066(19)	1	4*i*	0.019(8)
**Cs1**	0.5	0	0.5	1	2*c*	0.0375(29)

Table S8: Refined lattice parameters, atomic positions and thermal parameters for CsI. Corresponding diffraction data shown in Figure S7.

CsI					
**wR**	3.41%	**R**	2.29%	**χ^2**	2.14
**2ϑ range**	10-64.9967°	**Radiation**	Cu Kα, 1.5402Å	**Temp**	295 K
**Formula**	CsI	**Z**	1	**wt.%**	2.962(66)
a **(Å)**	4.56603(20)	***V*** **(Å³)**	95.195(12)	**S.G.**	$Pm\bar{3}m$

**Atom**	**x**	**y**	**z**	**frac**	**Wyckoff**	**Uiso**
**Cs1**	0	0	0	1	1*a*	0.002
**I1**	0.5	0.5	0.5	1	1*b*	0.02

Table S9: Comparison of material composition measurements by Rietveld refinement, energy-dispersive X-ray spectroscopy and inductively coupled plasma mass spectrometry.

Compound	Rietveld	EDX	ICP/MS
λ-V_2O_5	V_2O_5	V_2O_5	$Cu_{0.003}V_2O_5$
λ-$Li_1V_2O_5$	-	-	$Li_{1.021}V_2O_5$
λ-$Na_{0.833}V_2O_5$	$Na_{0.833}V_2O_5$	$Na_{0.92}V_2O_5$	$Na_{0.848}V_2O_5$
λ-$K_{0.667}V_2O_5$	$K_{0.667}V_2O_5$	$K_{0.65}V_2O_5$	$K_{0.628}V_2O_5$
λ-$Rb_{0.469}V_2O_5$	$Rb_{0.469}V_2O_5$	$Rb_{0.47}V_2O_5$	$Rb_{0.490}V_2O_5$
λ-$Cs_{0.5}V_2O_5$	$Cs_{0.5}V_2O_5$	$Cs_{0.51}V_2O_5$	$Cs_{0.548}V_2O_5$

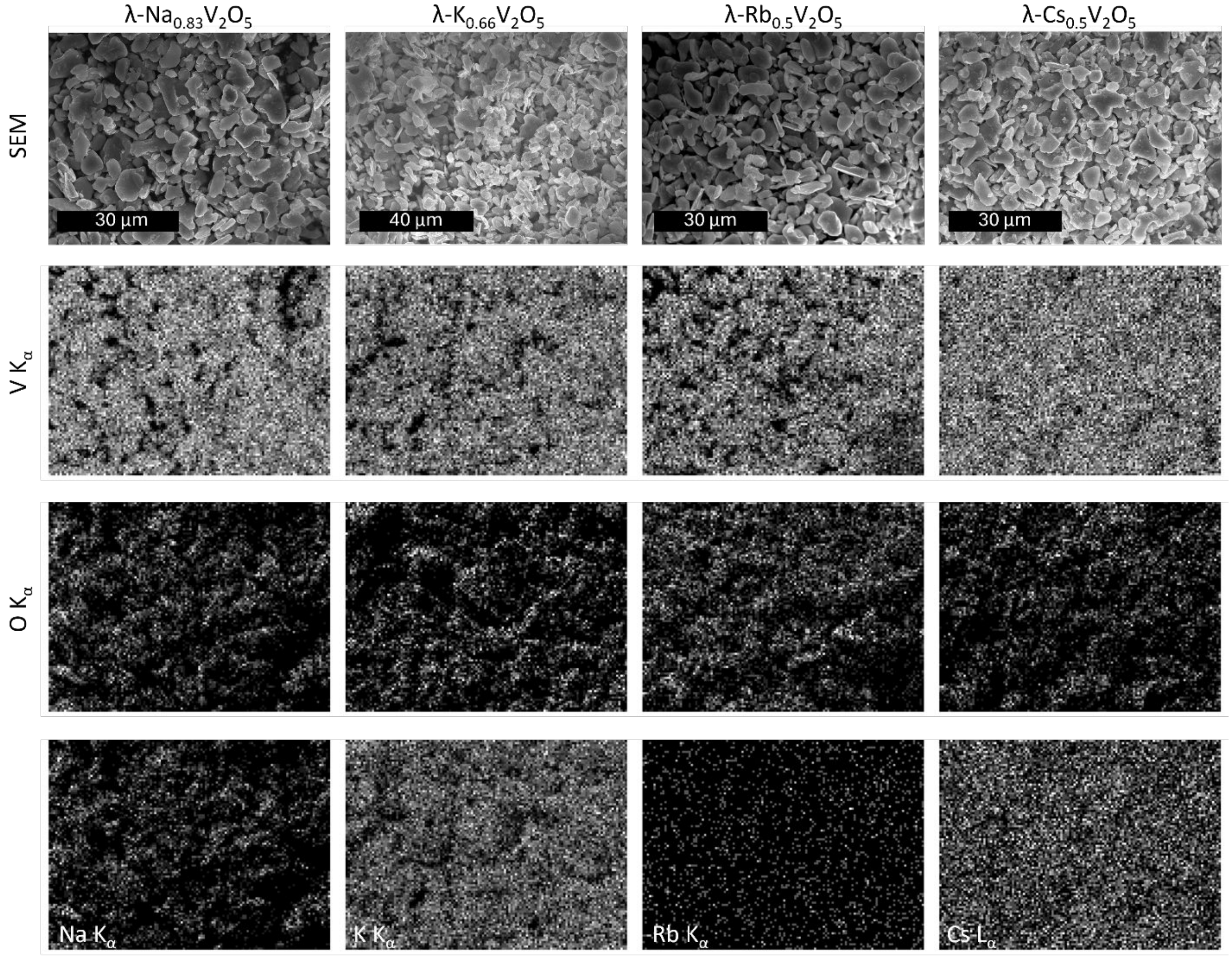

Figure S8: SEM images and EDX maps for λ-$Na_{0.83}V_2O_5$, λ-$K_{0.66}V_2O_5$, λ-$Rb_{0.5}V_2O_5$, and λ-$Cs_{0.5}V_2O_5$. SEM images indicate that all samples share the parent material's irregular plate

morphology attesting to topochemical ion insertion. Homogenous elemental compositions are mapped across different particles.

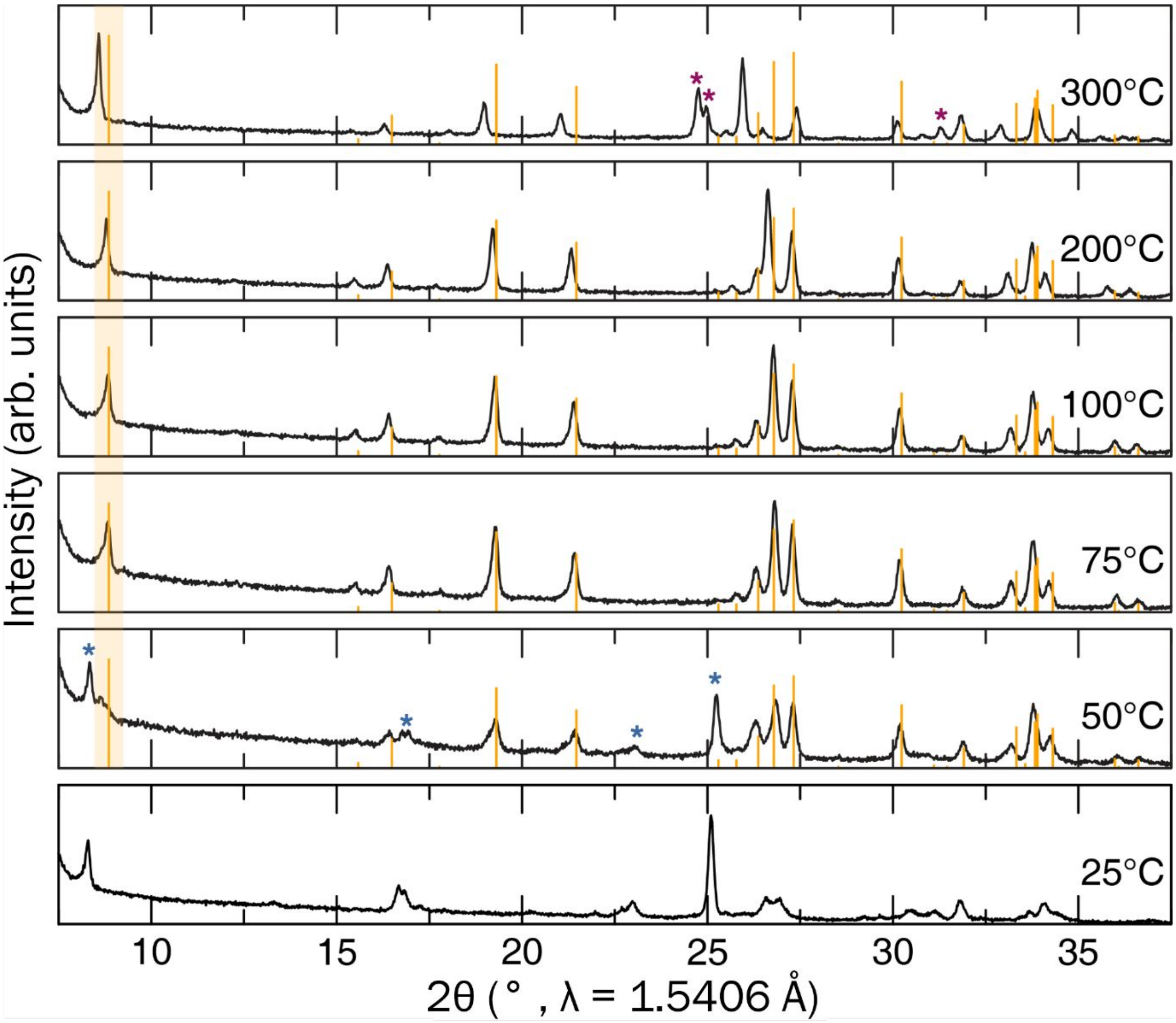

Figure S9: Temperature-dependent powder XRD patterns of hydrated λ-$Cs_{0.5}V_2O_5$. λ-$Cs_{0.5}V_2O_5$ hydrated in air for 7 days, then annealed for 1 h and measured for 2 h at each indicated temperature. At 25˚C, two distinct reflections at $2\theta = 8.3^{\circ}$ and 25.2 ˚ are indexed to the 001 and 003 reflections of the hydrated phase, respectively, and indicate relatively uniform interlayer distances. Upon heating to 50˚C, the material largely reverts to its original structure (yellow vertical lines), although some hydrated material remains (reflections denoted by blue asterisks). Upon heating at 75˚C the material has been completely dehydrated; narrowing of the observed reflections denotes increased crystallinity. Apart from thermal lattice expansion, the structure persists up to approx. 300˚C where unidentified decomposition products (purple asterisks) begin to appear.

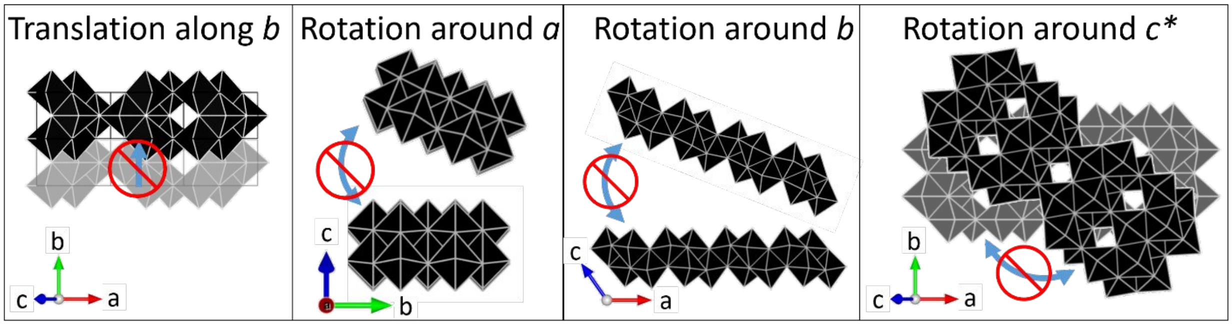

Figure S10: Crystallographic factors that constrain the stacking geometry of λ-$M_xV_2O_5$ materials to two structural degrees of freedom. Because of the C-centering symmetry operation, translation along ***b*** by ***b***/2 is equivalent to translation along ***a*** by ***a***/2. Smaller translation increments than ***b***/2 (i.e. smaller than half of one VO_6 octahedron along ***b***) break the local mirror symmetry preferred by spherical cations. Curiously, δ-$Sr_{0.5}V_2O_5$ has been reported to exhibit such a triclinic distortion, albeit exceedingly small.[1] Arbitrary rotation about any crystallographic axis breaks the translation symmetry necessary for crystalline order. However, 180˚ rotation of alternating layers about ***a*** or ***c****** generates the ρ-$M_zV_2O_5$ structure.[2]

Table S10: Published structure parameters for double-layered V_2O_5 bronzes.

All materials synthesized in this work are denoted by the λ prefix. Structure solutions have been published for λ_1-V_2O_5 (as λ-V_2O_5), λ-$Li_1V_2O_5$, and λ-$Rb_{0.5}V_2O_5$ (as $Rb_{0.5}V_2O_5$). Note that at least one structure is known in which alternating V_2O_5 layers are mirror-imaged; these materials fall outside the scope of the discussion in the main text but can be described in the proposed nomenclature by the use of a minus sign to indicate the mirroring of the polygon orientation (e.g. K^+ in ρ-$K_{0.5}V_2O_5$ inhabits an R-R site). To facilitate comparison, the lattice parameters of several structures have been modified as denoted in the Idealization column, with the details of each modification indicated by Roman numerals as follows:

i: In order to maintain consistent orientation of V_2O_5 lattices with respect to a right-handed coordinate system, a unit cell was adopted with a β angle < 90˚.

ii: To highlight monotonic relationship between Σ and Δ, an increment of a was added to $c \times cos(\beta)$, corresponding to translation along a by one unit cell to generate an equivalent setting.

iii: This structure is reported as triclinic, with $\alpha = 89.989˚$ and $\gamma = 90.029˚$. To facilitate comparison with other structures, this unit cell was approximated as monoclinic by setting $\alpha = \gamma = 90˚$.

iv: This structure is reported with a large unit cell and can be considered a supercell resulting primarily from ordered guest ion site occupancy. To facilitate comparison with other structures, the reported unit cell was divided to a corresponding size.

Material	*a* (Å)	*b* (Å)	*c* (Å)	α (˚)	β (˚)	γ (˚)	V (Å³)	Σ (Å)	Δ (Å)	Idealization	Reference
λ_1-V_2O_5	11.6524	3.6223	9.6091	90	59.348	90	348.92	8.27	4.89	i	3
λ_2-V_2O_5	11.65973	3.62213	8.85126	90	70.504	90	352.38	8.34	2.95	i	this work
λ-$Li_1V_2O_5$	11.69479	3.6816	10.24152	90	125.0618	90	360.94	8.38	5.81	ii	this work
λ-$Na_{0.8}V_2O_5$	11.8128	3.67124	9.336	90	70.071	90	380.63	8.78	3.18	i	this work
λ-$K_{0.66}V_2O_5$	11.703	3.6635	9.5904	90	101.672	90	402.68	9.39	-1.94		this work
λ-$Rb_{0.5}V_2O_5$	11.5469	3.67849	9.6913	90	100.9067	90	404.20	9.52	-1.83		this work
λ-$Cs_{0.5}V_2O_5$	11.5839	3.70349	10.1057	90	100.549	90	426.22	9.93	-1.85		this work
λ-$Li_1V_2O_5$	11.684	3.6786	10.203	90	124.884	90	359.73	8.37	5.85		3
$Rb_{0.5}V_2O_5$	11.596	3.6908	9.723	90	100.93	90	408.58	9.55	-1.84		4
δ-$Ag_{0.84}V_2O_5$	11.77	3.6748	8.7394	90	90.537	90	377.98	8.74	-0.08		5
ε-$Cu_{0.95}V_2O_5$	11.765	3.6943	8.9712	90	68.427	90	362.61	8.34	3.30	i	5
τ-$Cu_{0.43}Ag_{0.45}V_2O_5$	11.757	3.6942	9.463	90	65.38	90	373.64	8.60	3.94	i	5
δ-$Na_{0.56}V_2O_5$	11.663	3.6532	8.92	90	89.09	90	380.01	8.92	0.14	i	6
δ-$Sr_{0.5}V_2O_5$	23.53	7.3985	8.801	90	88.28	90	1531.45	8.80	0.26	i, iii, iv	1
δ-$Ca_xV_2O_5$	11.805	3.709	9.27	90	101.87	90	397.21	9.07	-1.91		7
δ-$Tl_{0.48}V_2O_5$	11.598	3.684	9.6293	90	100.826	90	404.11	9.46	-1.81		8
δ-$Pb_{0.5}V_2O_5$	11.716	3.6987	8.8186	90	88.280	90	381.81	8.815	0.26	i, iv	9
δ-$K_{0.5}V_2O_5$	11.688	3.668	9.505	90	87.76	90	407.18	9.50	0.37	i	10

**Inverted Structures**											
$K_{0.5}V_2O_5$	3.6784	11.612	18.6332	90	90	90	795.888	9.32	N/A		[11]
ρ-$K_{0.5}V_2O_5$	3.674	11.607	18.67	90	90	90	796.16	9.34	N/A		[2]
**Hydrated/Dehydrated Materials**											
δ-$Ni_{0.22}V_2O_5$•$0.94H_2O$	11.756	3.649	10.364	90	84.97	90	442.8	10.32	0.91	i	[12]
δ-$Ca_{0.24}V_2O_5$•$1H_2O$	11.692	3.564	10.986	90	105.42	90	452.0	10.59	-2.92		[12]
δ-$[Ni(H_2O)_4]_{0.2475}V_2O_5$ •$0.147H_2O$	11.753	3.648	10.372	90	84.913	90	442.935	10.33	0.92	i	[13]
$Na_{0.37}V_2O_5$•$1.03H_2O$	No structure solution							10.85			[14]
$K_{0.32}V_2O_5$•$0.91H_2O$	No structure solution							10.88			[14]
$Rb_{0.30}V_2O_5$•$0.76H_2O$	No structure solution							10.85			[14]
$Cs_{0.34}V_2O_5$•$0.67H_2O$	No structure solution							10.98			[14]
$(NH4)_{0.28}V_2O_5$•$1.02H_2O$	No structure solution							10.99			[14]
$Na_{0.37}V_2O_5$	No structure solution							9.01			[14]
$K_{0.32}V_2O_5$	No structure solution							9.49			[14]
$Rb_{0.30}V_2O_5$	No structure solution							9.80			[14]
$Cs_{0.34}V_2O_5$	No structure solution							10.26			[14]
$(NH_4)_{0.28}V_2O_5$	No structure solution							9.78			[14]

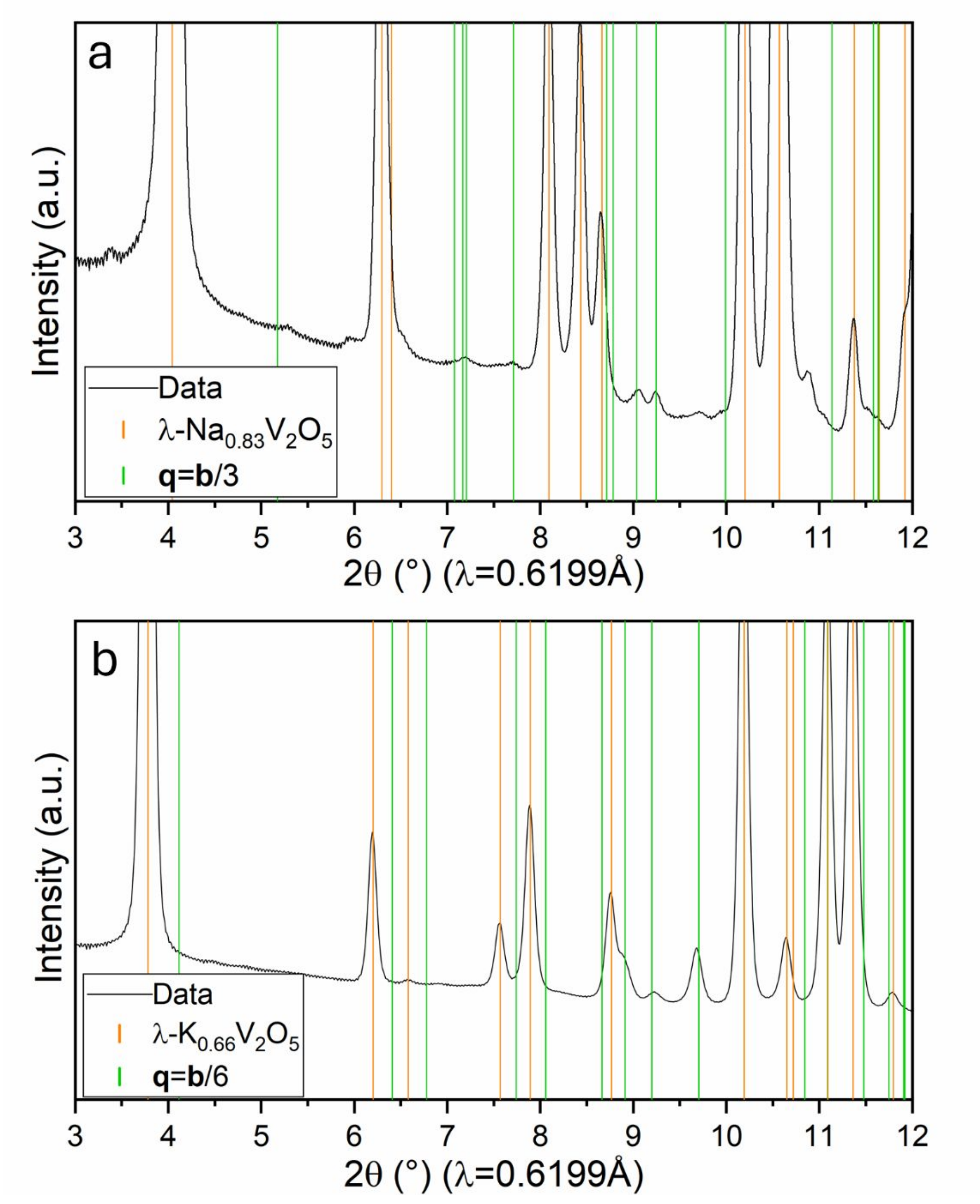

Figure S11: Modulation satellites in λ-$Na_{0.83}V_2O_5$ and λ-$K_{0.66}V_2O_5$. Conventional reflections and modulation satellites marked by orange and green vertical lines, respectively, in powder XRD patterns. (a) in λ-$Na_{0.83}V_2O_5$, a modulation vector $q = (0,{}^1/_3,0)$ corresponds roughly to 5 Na ions in 6 Na1 sites and 5 Na ions in 12 Na2 sites as per a 1×3×1 supercell. (b) A modulation vector $q = (0,{}^1/_6,0)$ in λ-$K_{0.66}V_2O_5$ corresponds roughly to 8 K ions in 12 K1 sites and 8 K ions in 24 K2 sites as per a 1×6×1 supercell.

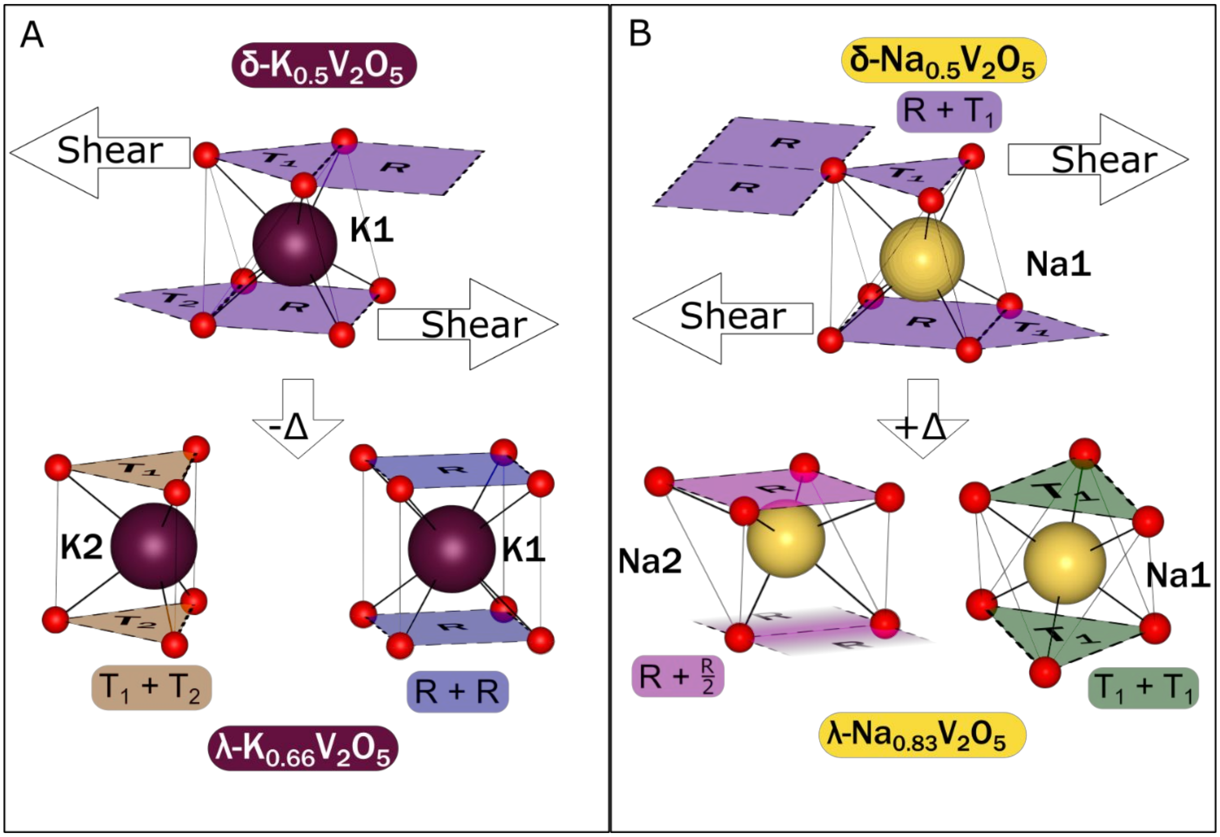

Figure S12: Shear transformations relating δ-$M_{0.5}V_2O_5$ and λ-$M_xV_2O_5$ structures. (A) M = K. Two R+T_1 sites transform into one R+R site and two T_1 + T_2 sites. In the orientation shown, leftward motion of the upper V_2O_5 slab leads to decreased Δ. **(B)** M = Na. Two R+T_1 sites transform into one T_1+T_1 site and two R+R/2 sites. Rightward motion of the upper slab leads to increased Δ. In each case, the λ structure contains more intercalation sites and thus admits higher guest ion concentrations than the δ structure.

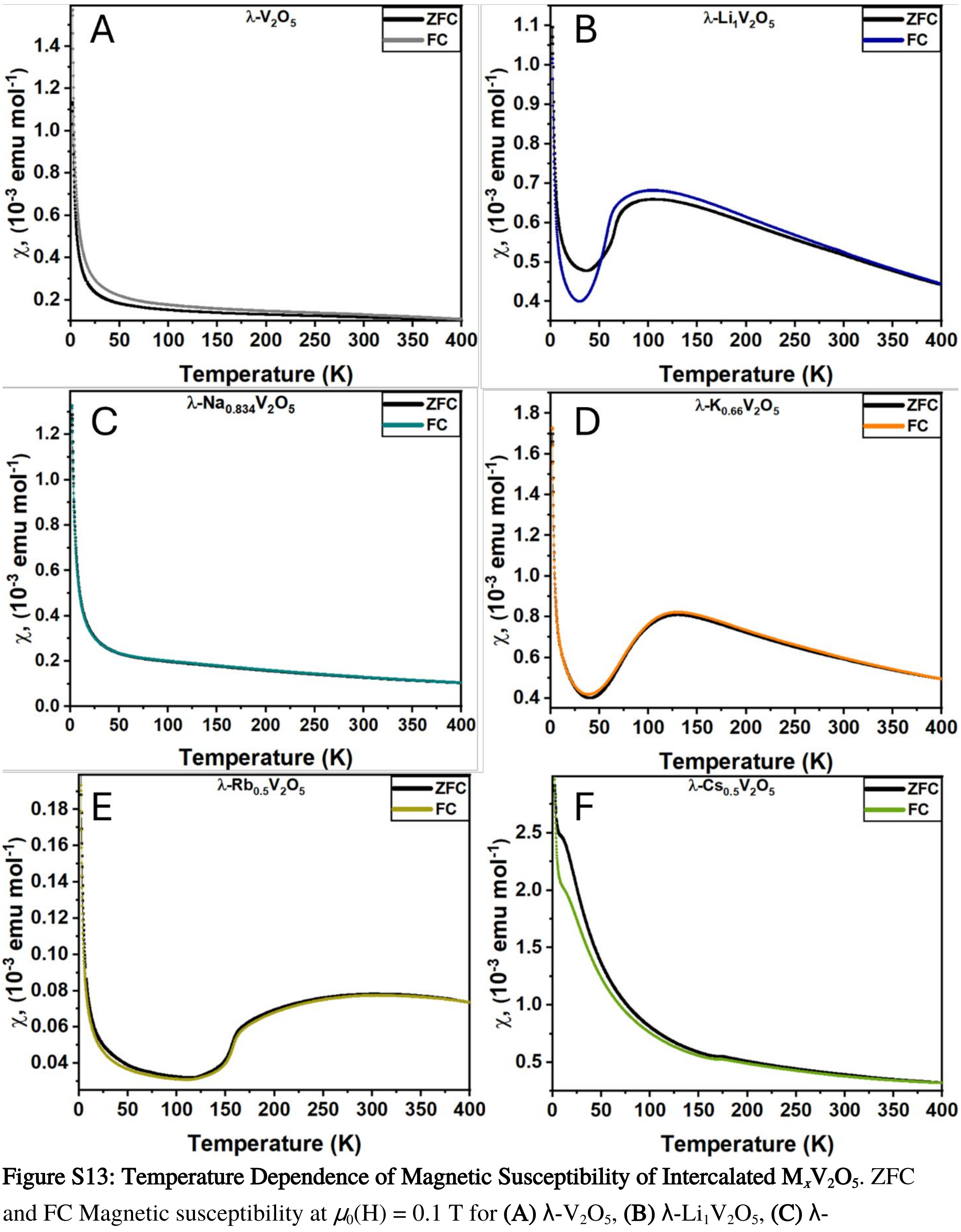

Figure S13: Temperature Dependence of Magnetic Susceptibility of Intercalated $M_xV_2O_5$. ZFC and FC Magnetic susceptibility at μ_0(H) = 0.1 T for **(A)** λ-V_2O_5, **(B)** λ-$Li_1V_2O_5$, **(C)** λ-$Na_{0.834}V_2O_5$, **(D)** λ-$K_{0.66}V_2O_5$, **(E)** λ-$Rb_{0.5}V_2O_5$, and **(F)** λ-$Cs_{0.5}V_2O_5$.

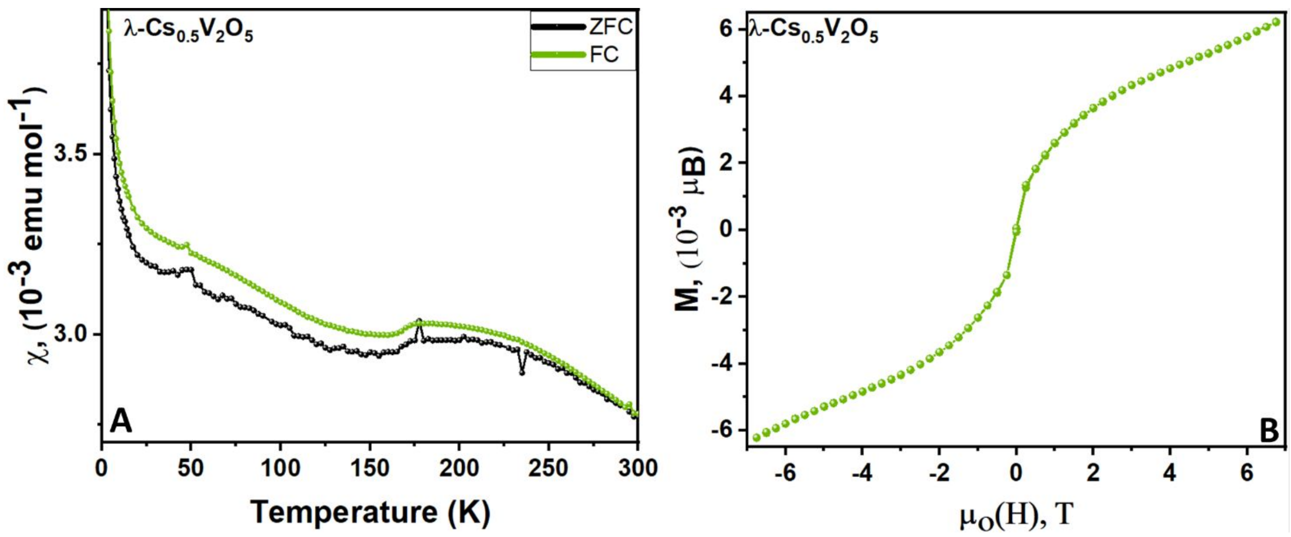

Figure S14: (A) ZFC and FC magnetic susceptibility of a dehydrated λ-$Cs_{0.5}V_2O_5$ specimen at μ_0(H) = 0.1 T measured under air-free conditions. (B) Magnetization versus magnetic field curve of λ-$Cs_{0.5}V_2O_5$ at 2 K. The magnetization behavior of λ-$Cs_{0.5}V_2O_5$ specimens is strongly modified by moisture absorption.

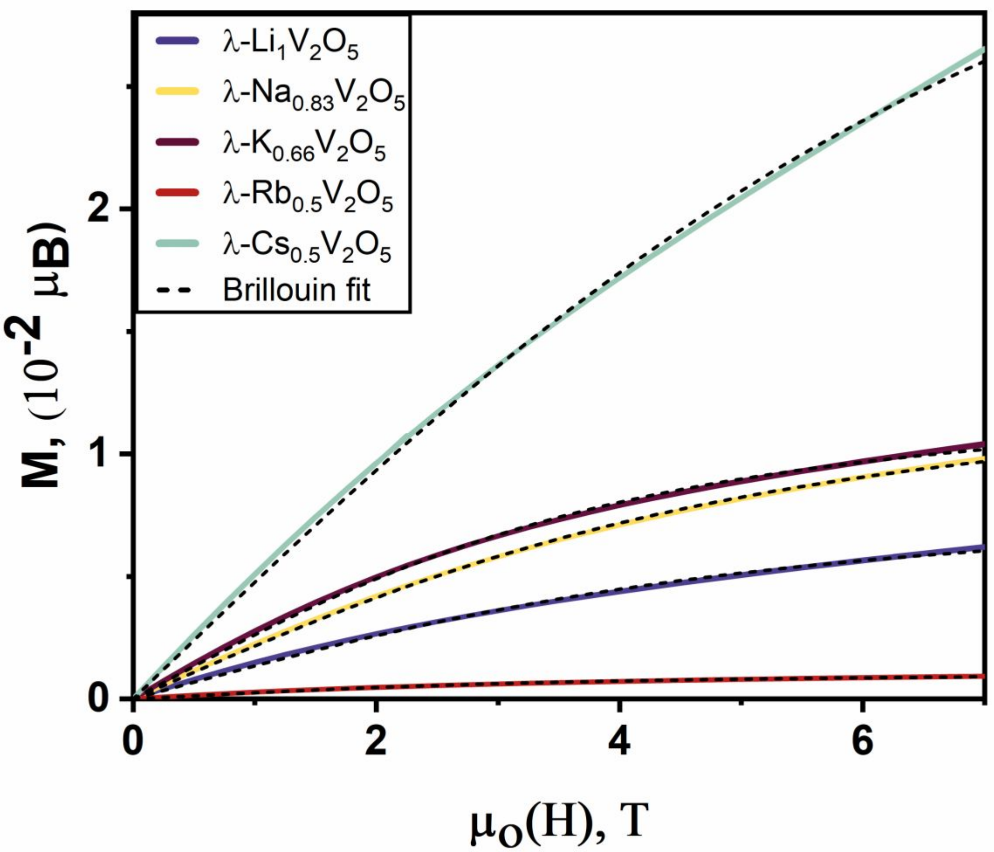

Figure S15: Field-dependent magnetization and Brillouin-function fit of Intercalated $M_xV_2O_5$. Fitting of magnetization vs magnetic field curve for the intercalated compounds using Brillouin-function fits. The fits yield saturation magnetization (M_{sat}) value of 2.0×10^{-2} μ_B for λ-$Li_1V_2O_5$, 1.1×10^{-2} μ_B for λ-$Na_{0.834}V_2O_5$, 2.2×10^{-2} μ_B for λ-$K_{0.66}V_2O_5$, 2.4×10^{-3} μ_B for λ-$Rb_{0.5}V_2O_5$, and 6.0×10^{-2} μ_B for λ-$Cs_{0.5}V_2O_5$. The black dashed line represents a fit using the Brillouin function.

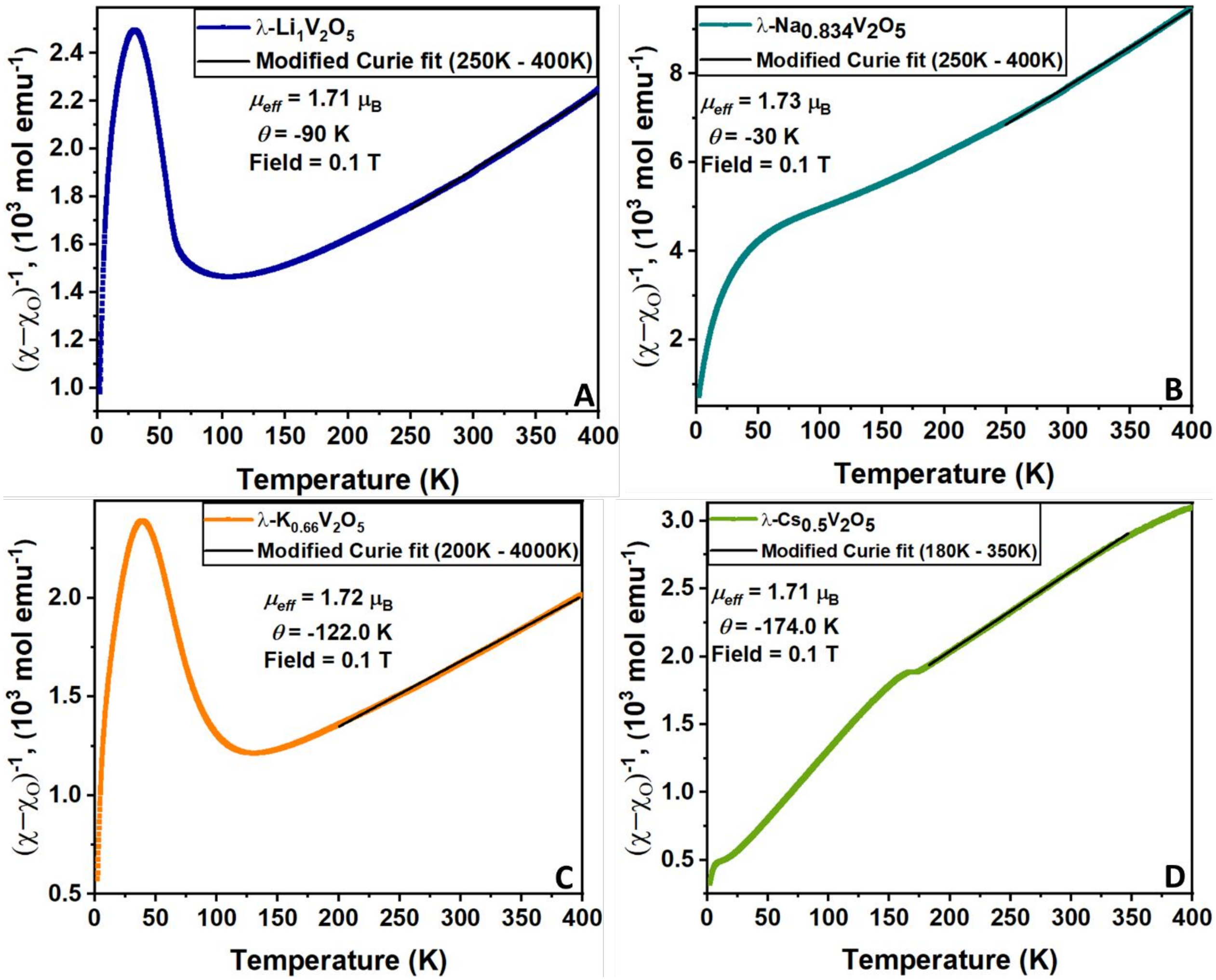

Figure S16: Inverse Magnetic Susceptibility of Intercalated $M_xV_2O_5$. Reciprocal plot of the magnetic susceptibility of **(A)** λ-$Li_1V_2O_5$, **(B)** λ-$Na_{0.834}V_2O_5$, **(C)** λ-$K_{0.66}V_2O_5$, and **(D)** λ-$Cs_{0.5}V_2O_5$. The relevant fit parameters obtained are presented within each panel.

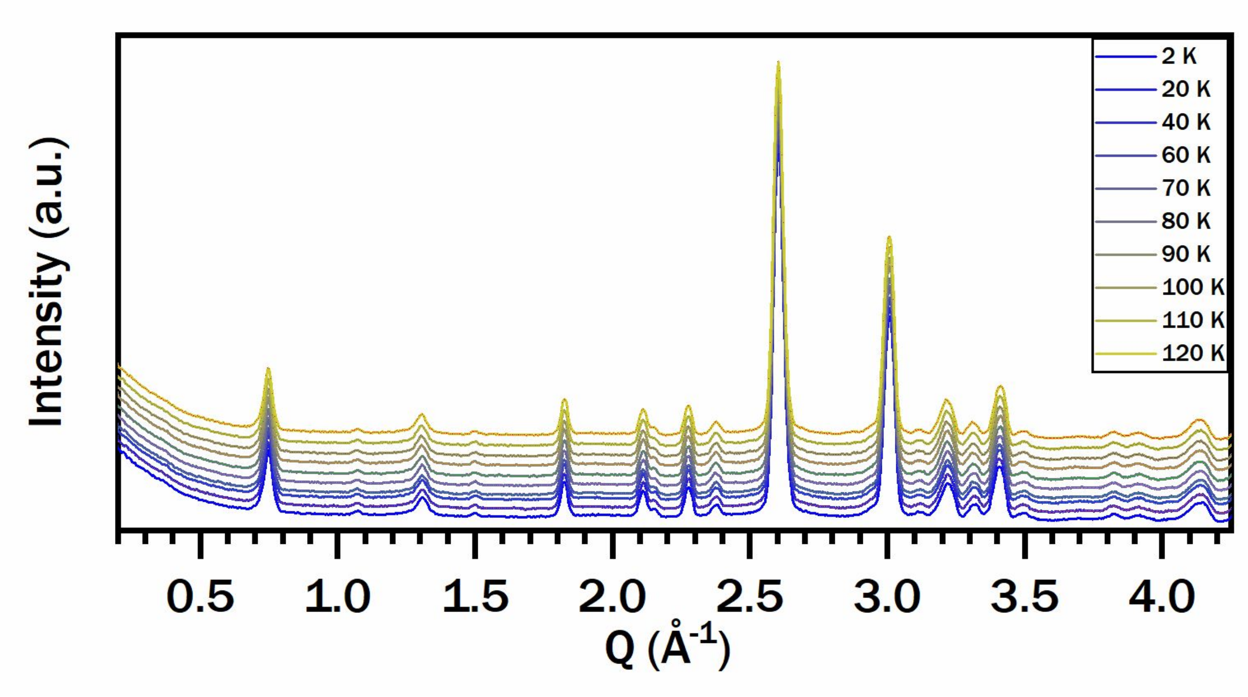

Figure S17: Temperature-dependent neutron diffraction patterns for λ-$Li_1V_2O_5$. No magnetic reflections appear upon cooling below the apparent transition temperature determined from magnetometry (89 K).

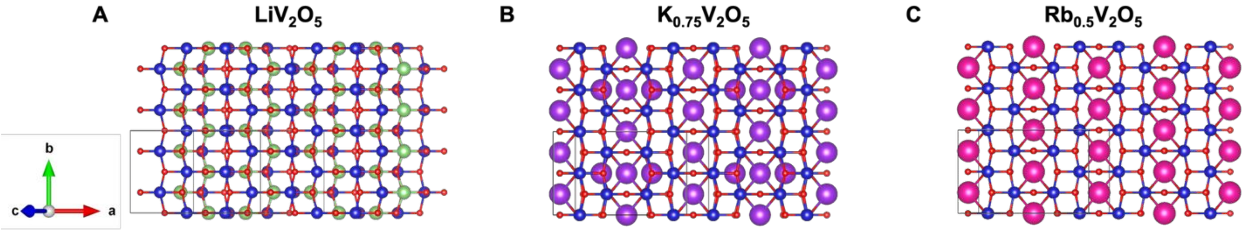

Figure S18. Atomic structures of intercalated V_2O_5. (A) LiV_2O_5, **(B)** $K_{0.75}V_2O_5$, and **(C)** $Rb_{0.5}V_2O_5$. The intercalated atoms, Li, K, and Rb, are shown in green, purple, and magenta, respectively. The rectangular outlines mark representative supercell regions.

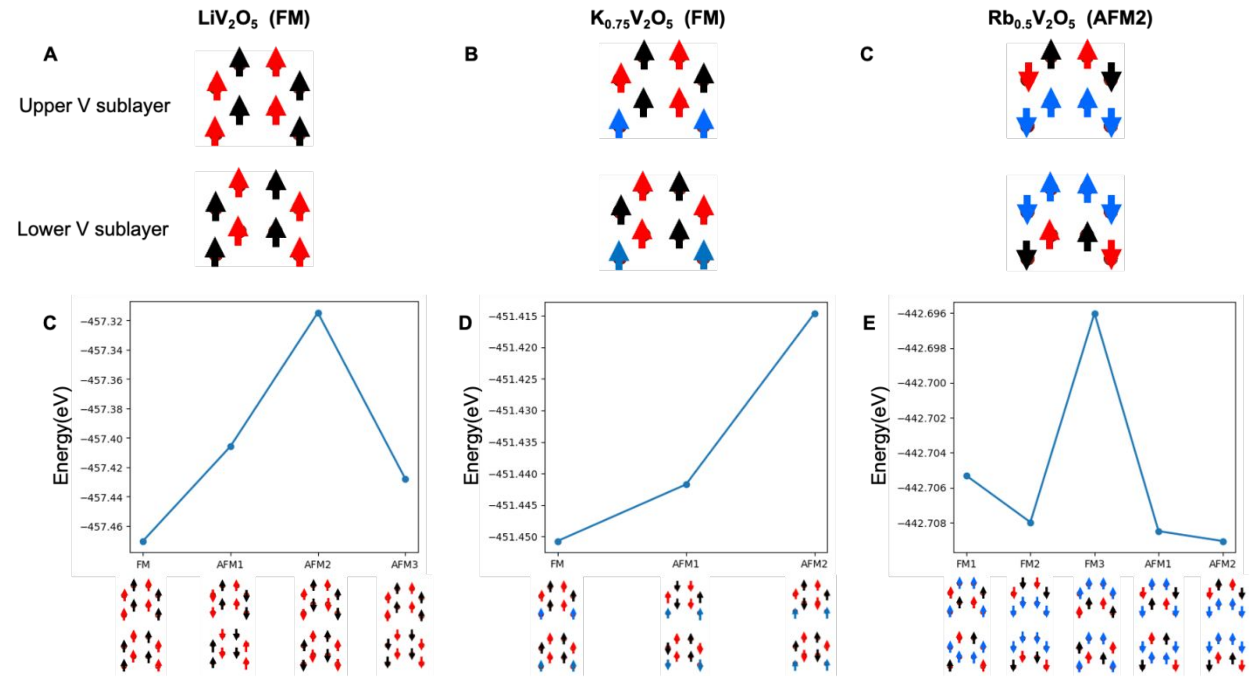

Figure S19. Magnetic configurations and corresponding energies of intercalated $M_xV_2O_5$. (A-C) The most stable magnetic configuration of LiV_2O_5, $K_{0.75}V_2O_5$, and $Rb_{0.5}V_2O_5$, respectively. **(D-F)** Total energies of different FM and AFM configurations for **(D)** LiV_2O_5, **(E)** $K_{0.75}V_2O_5$, and **(F)** $Rb_{0.5}V_2O_5$. In these three compounds, the black, red, and blue arrows denote magnetic moments of approximately 1 μB, 0.12 - 0.14 μB, and 0.03 - 0.09 μB, respectively.

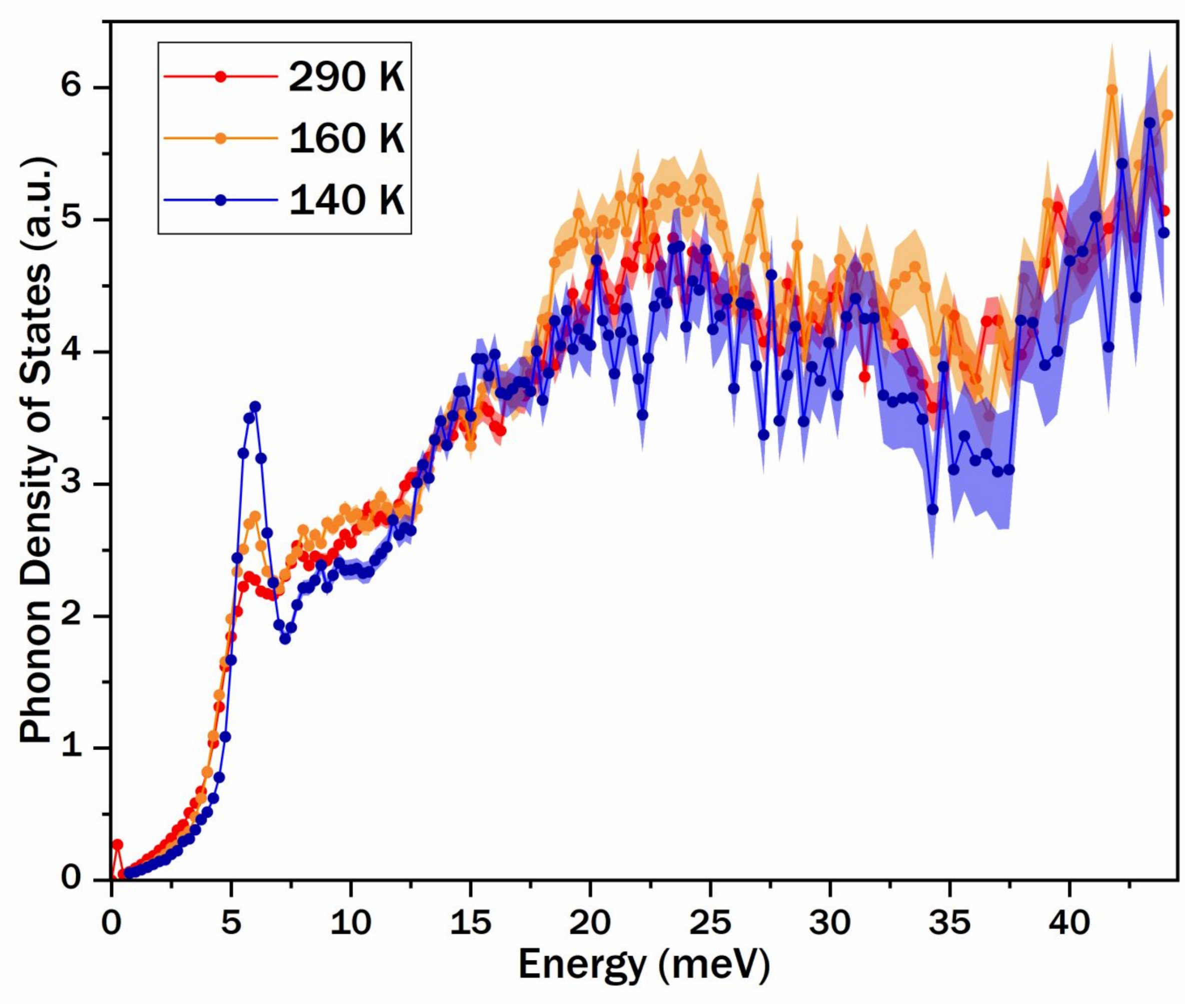

Figure S20: Temperature-dependent phonon density of states for ε-$Cu_{0.9}V_2O_5$. Calculated from inelastic neutron scattering data. The region between 0 meV and 15 meV is shown in **Figure 6D** in the main text.

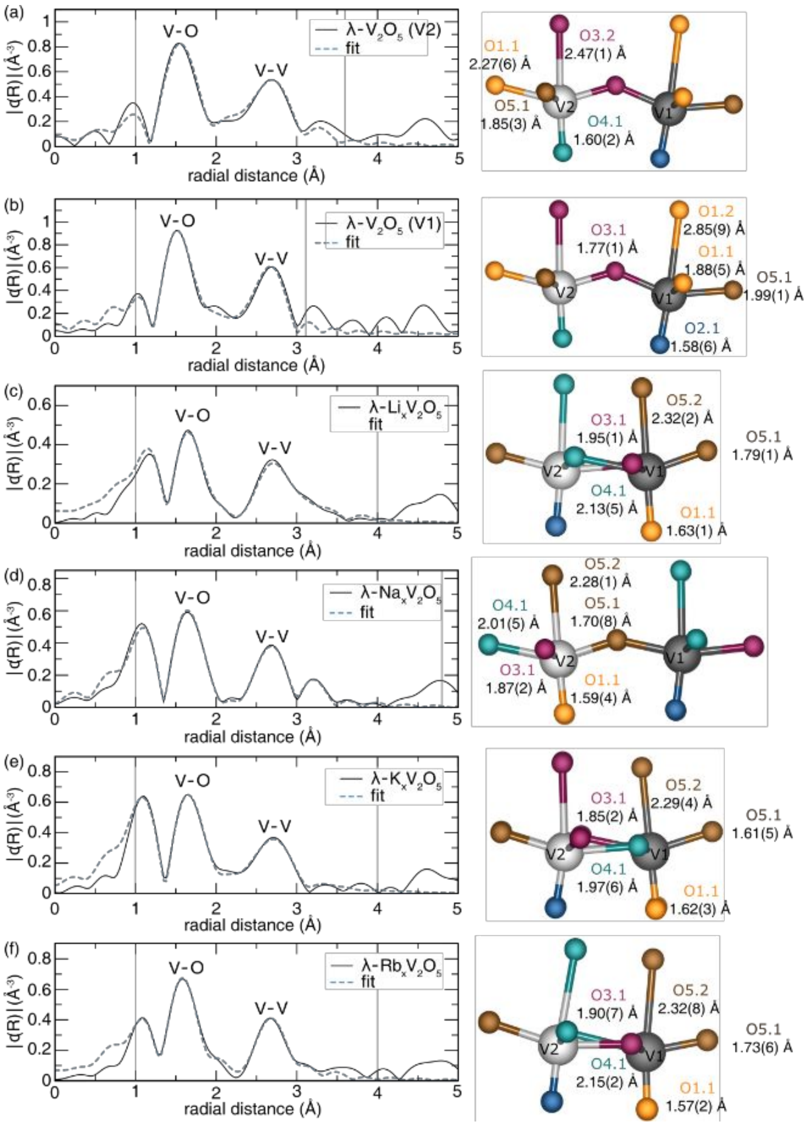

Figure S21. EXAFS fitting results for λ-V_2O_5 from the perspective of (a) V2 and (b) V1, (c) λ-$Li_xV_2O_5$ (V1), (d) $Na_xV_2O_5$ (V2), (e) $K_xV_2O_5$ (V1), and (f) $Rb_xV_2O_5$ (V1). The local structure parameters of λ-V_2O_5 and intercalated λ-$M_xV_2O_5$ (M = Li, Na, K, Rb) were determined by fitting the EXAFS oscillations starting with structural models obtained from powder X-ray diffraction. The fitting window is depicted by the grey lines. For the present analysis, only the V–O and V–V paths were employed. The amplitude reduction factor, S_0^2, and degeneracy, N, of the pristine and substituted λ-$M_xV_2O_5$ (M = Li, Na, K, Rb) were fixed, whereas the radial distances, R, mean squared radial displacement, σ^2, and edge energy, E_0, were varied to obtain the best fit. The corresponding values are provided in **Tables S11 (λ-V_2O_5)**, **S12 (λ-$Li_xV_2O_5$)**, **S13 (λ-$Na_xV_2O_5$)**,

S14 (λ-$K_xV_2O_5$), and **S15 (λ-$Rb_xV_2O_5$)**. The resulting V—O bond lengths are shown to the right of each fitting and indicate that the V—O bonds become more symmetrical upon cation insertion, relative to the pristine λ-V_2O_5.

Table S11. Results of EXAFS fitting of pristine λ-V_2O_5 from the perspective of V1 and V2.

Sample	Path	N	*R* (Å)	σ^2 (Å^2)	S_0^2	ΔE_0 (eV)	*R*-factor
	V1-O2.1	1	1.586	0.00013			
	V1-O3.1	1	1.771	0.00013			
λ-V_2O_5 (V1)	V1-O1.1	2	1.886	0.00013	0.75	6.055	1.75%
	V1-O5.1	1	1.991	0.00013			
	V1-O1.2	1	2.859	0.00013			
	**V1-V(2)**	**2**	**3.044**	0.00323			
	V2-O4.1	1	1.603	0.00256			
	V2-O3.1	1	1.934	0.00256			
	V2-O5.1	2	1.853	0.00256			
λ-V_2O_5 (V2)	V2-O1.1	1	2.276	0.00256	0.75	0.682	1.43%
	V2-O3.2	1	2.471	0.00256			
	V2-V1	2	3.030	0.00555			
	V2-V2	2	3.403	0.00555			
	V2-V2.1	2	3.594	0.00555			

Table S12. Results of EXAFS fitting of $Li_xV_2O_5$.

Sample	Path	N	*R* (Å)	σ^2 (Å^2)	S_0^2	ΔE_0 (eV)	*R*-factor
	V1-O2.1	1	1.631	0.00218			
	V1-O5.1	1	1.791	0.00218			
	V1-O3.1	2	1.950	0.00218			
	V1-O4.1	1	2.136	0.00218			
	V1-O5.2	1	2.322	0.00218			
$Li_xV_2O_5$	**V1-V2**	**2**	**3.017**	0.01056	1.00	2.717	1.03%
	V1-V1	1	3.318	0.01056			
	V1-V2.2	1	3.141	0.01056			
	V1-O2.1	2	3.262	0.00218			
	V1-V1.2	2	3.816	0.01056			
	V1-O3.2	2	3.950	0.00218			

Table S13. Results of EXAFS fitting of $Na_xV_2O_5$.

Sample	Path	N	*R* (Å)	σ² (Å²)	S_0^2	ΔE_0 (eV)	*R*-factor
	V2-O1.1	1	1.595	0.00087			
	V2-O5.1	1	1.708	0.00087			
	V2-O3.1	2	1.872	0.00087			
	V2-O4.1	1	2.015	0.00087			
$Na_xV_2O_5$	V2-O5.2	1	2.281	0.00087	0.768	0.302	3.02%
	**V2-V1**	**2**	**3.003**	0.00943			
	V2-V2	1	3.292	0.00943			
	V2-V1.3	1	3.615	0.00943			
	V2-O2.1	2	3.277	0.00087			

Table S14. Results of EXAFS fitting of $K_xV_2O_5$.

Sample	Path	N	*R* (Å)	σ² (Å²)	S_0^2	ΔE_0 (eV)	*R*-factor
	V1-O1.1	1	1.624	0.00433			
	V1-O5.1	1	1.616	0.00433			
	V1-O3.1	2	1.852	0.00433			
	V1-O4.1	1	1.977	0.00433			
$K_xV_2O_5$	V1-O5.2	1	2.294	0.00433	1.00	-4.84	1.30%
	**V1-V2**	**2**	**2.996**	0.00991			
	V1-V1	1	3.211	0.00991			
	V1-O2.1	3	3.386	0.00433			
	V1-V1.2	2	3.334	0.00991			

Table S15. Results of EXAFS fitting of $Rb_xV_2O_5$.

Sample	Path	N	*R* (Å)	σ² (Å²)	S_0^2	ΔE_0 (eV)	*R*-factor
	V1-O1.1	1	1.573	0.00135			
	V1-O5.1	1	1.737	0.00135			

	V1-O3.1	2	1.908	0.00135			
	V1-O4.1	1	2.152	0.00135			
$Cs_xV_2O_5$	V1-O5.2	1	2.329	0.00135	1.00	-6.104	1.62%
	**V1-V2**	**2**	**2.977**	0.00896			
	V1-V1	1	3.175	0.00896			
	V1-O2.1	2	3.180	0.00135			
	V1-V2.2	2	3.382	0.00896			

Supporting References

(1) Kato, K.; Kanke, Y.; Oka, Y.; Yao, T. Überstruktur Des Strontiumvanadiumoxids, $Sr_{0,5}V_2O_5$: Ihre Zwillingsbildung, Gruppoid-Symmetrie Und Interpretation Als OD-Struktur. *Z. Für Krist. - Cryst. Mater.* **1998**, *213* (7–8), 399–405. https://doi.org/10.1524/zkri.1998.213.7-8.399.

(2) Savariault, J.-M.; Galy, J. Synthesis and Structural Investigation of a New Potassium Vanadium Oxide Bronze: ϱ-$K_{0.50}V_2O_5$. *J. Solid State Chem.* **1992**, *101* (1), 119–127. https://doi.org/10.1016/0022-4596(92)90207-C.

(3) Handy, J. V.; Andrews, J. L.; Perez-Beltran, S.; Powell, D. R.; Albers, R.; Whittaker-Brooks, L.; Bhuvanesh, N.; Banerjee, S. A "Li-Eye" View of Diffusion Pathways in a 2D Intercalation Material from Topochemical Single-Crystal Transformation. *ACS Energy Lett.* **2022**, *7* (6), 1960–1962. https://doi.org/10.1021/acsenergylett.2c00739.

(4) Yao, T.; Oka, Y.; Yamamoto, N. Layered Structures of Hydrated Vanadium Oxides. Part 5.—Single-Crystal Structure of $Rb_{0.5}V_2O_5$ and Phase Changes of Rubidium Intercalate. *J Mater Chem* **1996**, *6* (7), 1195–1198. https://doi.org/10.1039/JM9960601195.

(5) Rozier, P.; Dollé, M.; Galy, J. Ionic Diffusion Mastering Using Crystal-Chemistry Parameters: τ-$Cu_{1/2}Ag_{1/2}V_2O_5$ Structure Determination and Comparison with Refined δ-$Ag_xV_2O_5$ and ε-$Cu_xV_2O_5$. *J. Solid State Chem.* **2009**, *182* (6), 1481–1491. https://doi.org/10.1016/j.jssc.2009.03.017.

(6) Kanke, Y.; Kato, K.; Takayama-Muromachi, E.; Isobe, M. Structure of $Na_{0.56}V_2O_5$. *Acta Crystallogr. C* **1990**, *46* (4), 536–538. https://doi.org/10.1107/S0108270189008589.

(7) Kutoglu, A. Kristallstruktur Der Calcium-Vanadium-Bronze $Ca_xV^{4+}{}_{2x}V^{5+}{}_{2-2x}O_5$. *Z. Für Krist. - Cryst. Mater.* **1983**, *162* (1–4), 263–272. https://doi.org/10.1524/zkri.1983.162.14.263.

(8) Onoda, M.; Hasegawa, J. The Spin-Gap State and the Phase Transition in the δ-Phase $Tl_xV_2O_5$ Polaronic Bronze. *J. Phys. Condens. Matter* **2002**, *14* (19), 5045. https://doi.org/10.1088/0953-8984/14/19/325.

(9) Agbeworvi, G.; Zaheer, W.; Handy, J. V.; Andrews, J. L.; Perez-Beltran, S.; Jaye, C.; Weiland, C.; Fischer, D. A.; Balbuena, P. B.; Banerjee, S. Toggling Stereochemical Activity through Interstitial Positioning of Cations between 2D V_2O_5 Double Layers. *Chem. Mater.* **2023**, *35* (17), 7175–7188. https://doi.org/10.1021/acs.chemmater.3c01463.

(10) Oka, Y.; Yaob, T.; Yamamotoc, N. Layered Structures of Hydrated Vanadium Oxides Part 4.-Single-Crystal Structure and Phase Conversion of the Unhydrated Potassium Intercalate $K_{0.5}V_2O_5$. *J MATER CHEM* **1995**, *5* (9), 1423–1426. https://doi.org/10.1039/JM9950501423.

(11) Kanke, Y.; Kato, K.; Takayama-Muromachi, E.; Isobe, M.; Kosuda, K. Structure of $K_{0.5}V_2O_5$. *Acta Crystallogr. C* **1990**, *46* (9), 1590–1592. https://doi.org/10.1107/S0108270189014228.

(12) Oka, Y.; Yao, T.; Yamamoto, N. Crystal Structures of Hydrated Vanadium Oxides with δ-Type V_2O_5 Layers: δ-$M_{0.25}V_2O_5 \cdot H_2O$,M=Ca, Ni. *J. Solid State Chem.* **1997**, *132* (2), 323–329. https://doi.org/10.1006/jssc.1997.7467.
(13) Andrews, J. L.; Singh, S.; Kilcoyne, C.; Shamberger, P. J.; Sambandamurthy, G.; Banerjee, S. Memristive Response of a New Class of Hydrated Vanadium Oxide Intercalation Compounds. *MRS Commun.* **2017**, *7* (3), 634–641. https://doi.org/10.1557/mrc.2017.64.
(14) Yao, T.; Oka, Y.; Yamamoto, N. Layered Structures of Hydrated Vanadium Oxides. Part 1.—Alkali-Metal Intercalates $A_{0.3}V_2O_5 \cdot nH_2O$ (A = Na, K, Rb, Cs and NH_4). *J Mater Chem* **1992**, *2* (3), 331–336. https://doi.org/10.1039/JM9920200331.
